# Comparative profiling of stress granule clearance reveals differential contributions of the ubiquitin system

**DOI:** 10.26508/lsa.202000927

**Published:** 2021-03-09

**Authors:** Nazife Tolay, Alexander Buchberger

**Affiliations:** Department of Biochemistry, Biocenter, University of Würzburg, Würzburg, Germany

## Abstract

Stress granules (SGs) are cytoplasmic condensates containing untranslated mRNP complexes. They are induced by various proteotoxic conditions such as heat, oxidative, and osmotic stress. SGs are believed to protect mRNPs from degradation and to enable cells to rapidly resume translation when stress conditions subside. SG dynamics are controlled by various posttranslational modifications, but the role of the ubiquitin system has remained controversial. Here, we present a comparative analysis addressing the involvement of the ubiquitin system in SG clearance. Using high-resolution immunofluorescence microscopy, we found that ubiquitin associated to varying extent with SGs induced by heat, arsenite, H_2_O_2_, sorbitol, or combined puromycin and Hsp70 inhibitor treatment. SG-associated ubiquitin species included K48- and K63-linked conjugates, whereas free ubiquitin was not significantly enriched. Inhibition of the ubiquitin activating enzyme, deubiquitylating enzymes, the 26S proteasome and p97/VCP impaired the clearance of arsenite- and heat-induced SGs, whereas SGs induced by other stress conditions were little affected. Our data underline the differential involvement of the ubiquitin system in SG clearance, a process important to prevent the formation of disease-linked aberrant SGs.

## Abstract (precis)

This study shows that ubiquitin conjugates associate with various types of stress granules and that an active ubiquitin system is required for the efficient clearance of some types of stress granules.

## Introduction

Eukaryotic cells adapt to various environmental and biotic stresses by down-regulation of bulk translation and disassembly of polysomes. As a result, untranslated messenger ribonucleoprotein complexes (mRNPs) accumulate in the cytoplasm, where they recruit numerous additional proteins including RNA binding proteins (RBPs). Through a multivalent network of protein–protein, protein–RNA, and RNA–RNA interactions, these mRNPs condense into dynamic membrane-less organelles called stress granules (SGs) (Hyman et al, 2014; Protter & Parker, 2016; Mittag & Parker, 2018; Hofmann et al, 2021). When stress conditions eventually subside, SGs disassemble and release the stored mRNPs, thereby allowing bulk translation to recommence.

SGs are heterogeneous in structure, size and composition and contain hundreds of proteins which reside either in the stable SG core or in a highly dynamic shell surrounding it (Jain et al, 2016; Aulas et al, 2017; Markmiller et al, 2018; Youn et al, 2018). The SG core consists of RBPs with intrinsically disordered regions and/or prion-like low-complexity domains, such as G3BP1/2 (henceforth collectively called G3BP), UBAP2L, TIA-1, hnRNPA1, and FUS, which possess the capacity to undergo liquid–liquid phase separation (LLPS) and to drive SG formation in living cells (Gilks et al, 2004; Molliex et al, 2015; Patel et al, 2015; Kedersha et al, 2016; Guillen-Boixet et al, 2020; Sanders et al, 2020; Yang et al, 2020; Hofmann et al, 2021). Importantly, perturbations in cellular SG homeostasis (also referred to as “granulostasis”) have been linked to several degenerative disorders, including amyotrophic lateral sclerosis, frontotemporal dementia (FTD), and multisystem proteinopathy (MSP) (Taylor et al, 2016; Alberti et al, 2017; Wolozin & Ivanov, 2019). These diseases can be caused by mutant RBPs with increased LLPS propensities, by mutational impairment of proteins promoting normal SG disassembly, or by non-AUG–driven translation of dipeptide repeat polypeptides altering SG dynamics (Taylor et al, 2016; Alberti et al, 2017). All these aberrations promote the formation of SGs containing aggregation-prone RBPs that tend to fibrillize and are believed to function as seeds for pathogenic aggregates (Lin et al, 2015; Molliex et al, 2015; Patel et al, 2015). However, despite significant progress in elucidating the pathogenesis underlying these ageing-related disorders, the molecular mechanisms controlling granulostasis in health and disease are still incompletely understood.

In living cells, SG dynamics are not only governed by the material properties of RBPs and mRNAs that drive LLPS, but additionally by proteostasis factors and posttranslational modifications (PTMs). Among the former, Hsp70 chaperones play central roles in granulostasis. Impairment of Hsp70 function by pharmacological inhibition, siRNA-mediated depletion or stress-induced overload induces SG formation (Mazroui et al, 2007; Ganassi et al, 2016). Moreover, Hsp70 chaperones in concert with BAG3 and HSPB8 promote the disassembly of SGs, and failure to do so results in the formation of aberrant, fibrillization-prone SGs (Ganassi et al, 2016; Mateju et al, 2017). Among PTMs, the covalent modification of proteins with ubiquitin (Ub), referred to as ubiquitylation, is the most versatile PTM in eukaryotes and controls various aspects of eukaryotic cell biology (Komander & Rape, 2012; Akutsu et al, 2016). Ubiquitylation requires three enzymatic activities, E1 (Ub-activating enzyme), E2 (Ub conjugating enzyme), and E3 (Ub protein ligase) (Komander & Rape, 2012), resulting in the conjugation of target proteins with single Ub moieties (mono-ubiquitylation) or, more commonly, with Ub chains of different lengths and linkage types. Importantly, the type of Ub modification defines the downstream fate of the target proteins (Akutsu et al, 2016; Yau & Rape, 2016). For example, proteins modified with K48-linked Ub chains are typically targeted for degradation by the 26S proteasome, whereas K63-linked Ub chains mark proteins for non-proteasomal fates in cellular processes such as endolysosomal trafficking, autophagy and DNA repair. Ub modifications can be edited or removed by deubiquitylating enzymes (DUBs), which further increase the plasticity of protein ubiquitylation. In addition, many Ub-controlled cellular processes require the activity of the ATPase p97 (also known as VCP and Cdc48), which unfolds and/or segregates ubiquitylated proteins to feed them into their designated downstream pathways (Buchberger et al, 2015; Bodnar & Rapoport, 2017; van den Boom & Meyer, 2018).

Various lines of evidence link the Ub system with granulostasis. The DUB USP10 binds G3BP, localizes to SGs and regulates SG formation, even though the exact role of its DUB activity in this process remains controversial (Kedersha et al, 2016; Nostramo & Herman, 2016; Sanders et al, 2020). Support for direct involvement of the Ub system in granulostasis comes from the frequently reported finding that Ub is present at SGs (Kwon et al, 2007; Seguin et al, 2014; Mateju et al, 2017; Turakhiya et al, 2018; Xie et al, 2018; Zhang et al, 2019). The Ub-binding proteasomal substrate adaptor UBQLN2 localizes to and negatively regulates the formation of SGs (Alexander et al, 2018; Dao et al, 2018). Moreover, several enzymes of the Ub system were found to associate with SGs, including the 26S proteasome (Turakhiya et al, 2018), p97 (Buchan et al, 2013; Turakhiya et al, 2018; Wang et al, 2019) and the DUBs USP5 and USP13 (Xie et al, 2018). Of note, all these enzymes promote the efficient clearance of SGs during recovery from stress treatments, collectively suggesting that the turnover of ubiquitylated proteins plays an important role in SG biology. Recently, however, two studies reported that SGs contain Ub species that are not conjugated to target proteins. Xie et al (2018) found that both protein-conjugated and free (“unanchored”) Ub chains associate with heat-induced SGs and need to be removed by USP13 and USP5, respectively, for efficient SG clearance (Xie et al, 2018). Markmiller et al (2019) claimed that Ub associated with arsenite-induced SGs primarily represents free, unconjugated mono-Ub. Using a pharmacological inhibitor of the Ub E1 enzyme, these authors further reported that active protein ubiquitylation is dispensable for normal assembly and clearance of arsenite-induced SGs (Markmiller et al, 2019). In particular this latter report is difficult to reconcile with the previously suggested active role of the Ub system in SG turnover. A synopsis of the available data, however, is complicated by the fact that many studies are based on a relatively narrow set of experimental conditions regarding the SG-inducing stress treatments used and/or the analysis of Ub involvement.

Here, we present a comparative study that directly addresses the Ub state of different types of SGs and the role of the Ub system in their turnover. We show that Ub conjugates associate with various types of SGs and that active protein ubiquitylation and proteasomal degradation are required for the efficient clearance of some of them.

## Results

### Ubiquitin associates with different types of SGs

**Figure 1.**
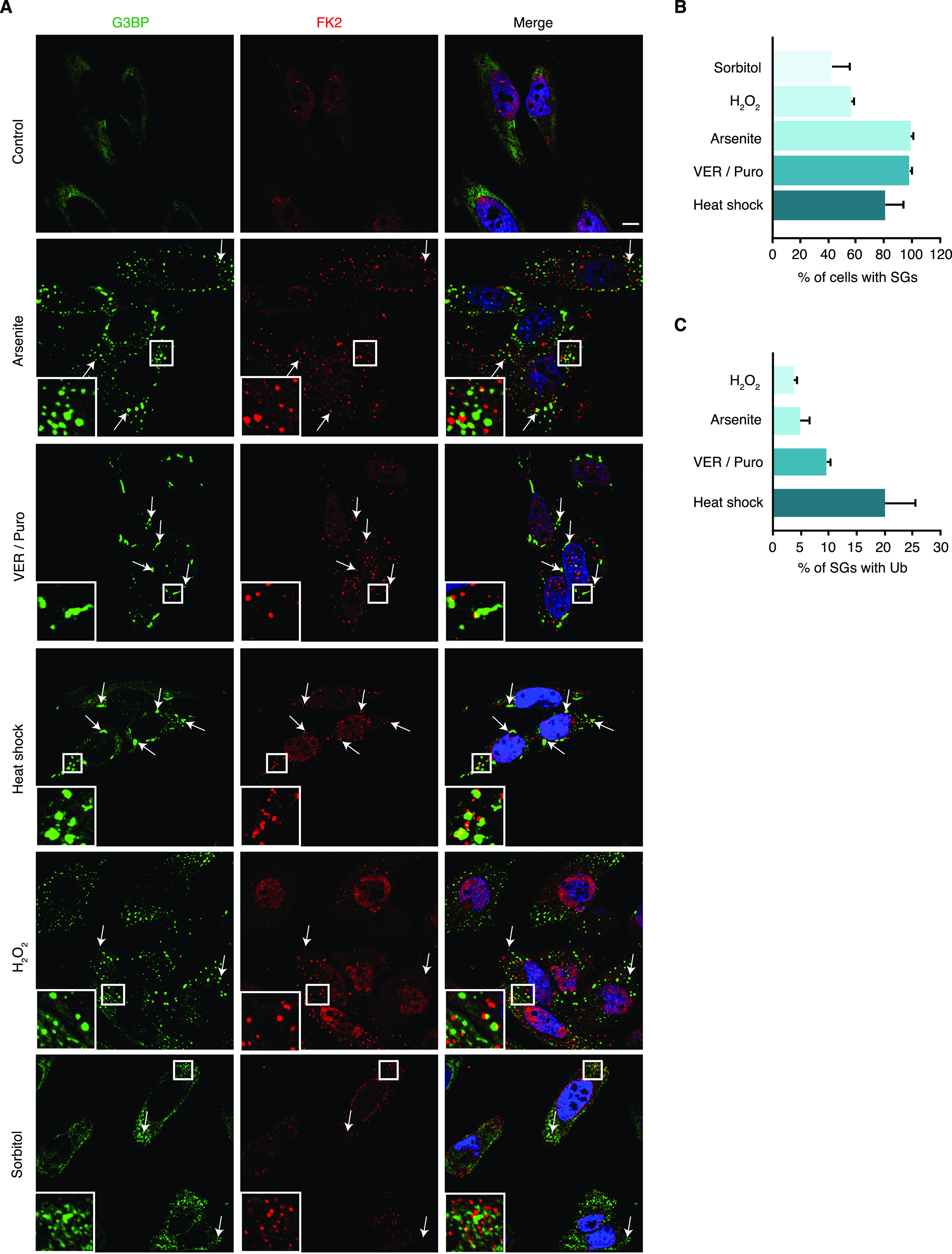
Ub associates with stress granules. **(A)** HeLa cells were subjected to treatment with arsenite (0.5 mM, 45 min), VER-155008 (40 μM) in combination with Puromycin (2.5 μg/ml, VER/Puro; 3 h), sorbitol (0.4 M, 4 h), H_2_O_2_ (1 mM, 2 h), and heat stress (43°C, 2 h) as indicated. The colocalization of G3BP and Ub (FK2) was visualized by confocal immunofluorescence microscopy. Representative FK2-positive SGs are marked by arrows or magnified in the inset. Scale bar, 10 μm. **(A, B)** Quantification of cells with SGs detected in (A); shown is the mean ± SD; n = 3 with ≥150 cells per condition. **(A, C)** Quantification of Ub-positive SGs detected in (A); shown is the mean ± SD; n = 2 with ≥1,000 SGs per condition.

We set out to systematically determine the association of Ub with different types of SGs using confocal immunofluorescence microscopy. For SG induction, we chose various established stressors including arsenite, oxidative stress (H_2_O_2_), heat shock, the Hsp70 inhibitor VER-155008 in combination with puromycin (VER/Puro), and hyperosmolarity (sorbitol) (Bounedjah et al, 2014; Kedersha et al, 2016; Aulas et al, 2017). Compared with H_2_O_2_, arsenite exerts its proteotoxic effects not only by inducing oxidative stress, but also by direct binding to proteins, as well as by inhibiting molecular chaperones and the 26S proteasome (Jacobson et al, 2012; Tamas et al, 2014; Tillotson et al, 2017). SGs were stained with an antibody against the core SG protein G3BP, and Ub conjugates were detected using the FK2 antibody, which recognizes Ub chains of various linkage types and mono-ubiquitylated proteins, but not free mono-Ub (Fujimuro & Yokosawa, 2005). Analyzing single confocal planes to exclude apparent signal overlap derived from signals in distinct focal planes, we were able to detect Ub-positive SGs under all five stress conditions tested (Fig 1A). Depending on the stress condition, between 45% and 100% of the cells contained SGs, with arsenite, VER/Puro and heat being the most efficient SG-inducing conditions (Fig 1B). Counting at least 1,000 SGs per condition in two biological replicates, we determined the percentage of SGs that overlapped with the FK2 signal according to the ComDet spot colocalization plugin for ImageJ/Fiji. Ub was found to associate with only few (ca. 5%) arsenite-induced SGs, about 10% of SGs induced by VER/Puro, and 20% of SGs induced by heat shock (Fig 1C). SGs induced by H_2_O_2_ were Ub-positive at very low frequency (<5%). Compared with the other stress conditions, SGs induced by hyperosmotic stress were smaller, less well defined, and only in some cells positive for Ub, precluding the quantitative analysis of FK2-positive SGs. Together, our data show that Ub associates with SGs induced by various stress conditions and that the frequency of association varies markedly between different types of SGs.

**Figure 2.**
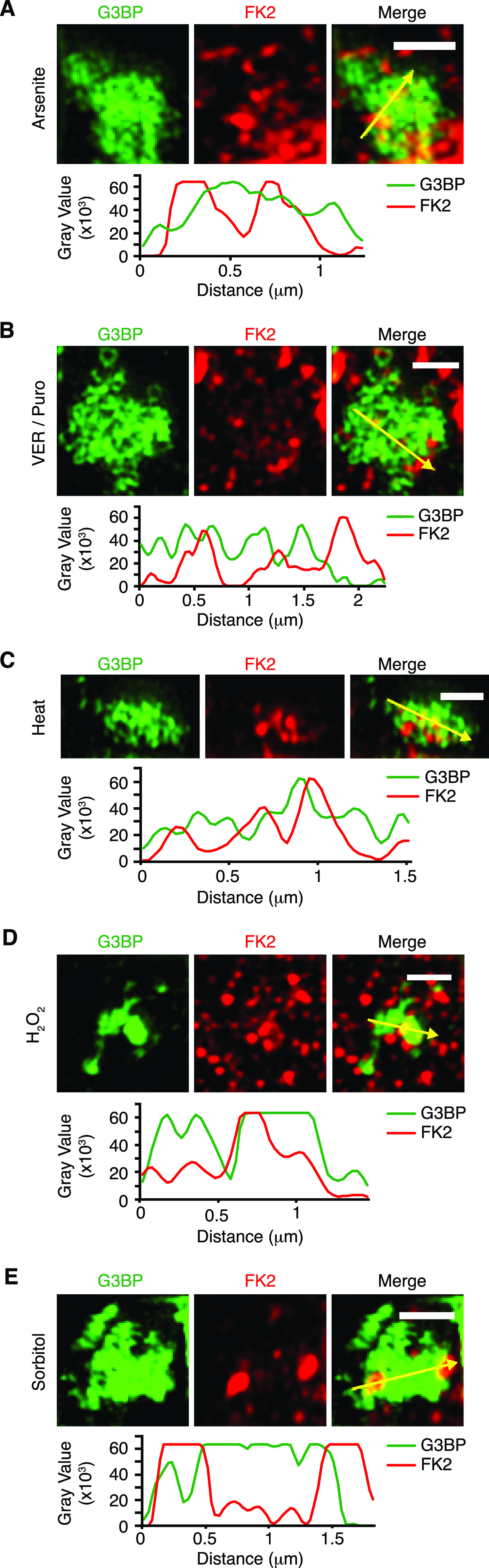
Ub mainly localizes to the periphery of stress granules. **(A, B, C, D, E)** Structured illumination microscopy of FK2-positive stress granules induced by arsenite (A), VER/Puro (B), heat stress (C), H_2_O_2_ (D), or sorbitol (E). Line profiles of G3BP and FK2 signals along the yellow arrows were generated using ImageJ. Scale bar, 1 μm.

The confocal microscopy images suggested that most Ub signals only partially overlap with the core SG protein, G3BP. To analyze the relative localization of Ub and G3BP at higher resolution, we performed structured illumination microscopy (SIM) on Ub-positive SGs induced by the different stress conditions (Fig 2A–E). Maximum intensity projections revealed that G3BP forms non-uniform structures with clusters of high G3BP concentration, in agreement with previous SIM analyses of SGs (Jain et al, 2016; Xie et al, 2018). Importantly, the FK2 signal was non-uniform as well and mostly found in the cavities and/or the periphery of the G3BP structures under all five stress conditions, with some regions of overlap with G3BP. These results indicate that Ub mainly localizes to the dynamic shell rather than the stable core of SGs.

### SG-associated ubiquitin chains predominantly represent conjugates

**Figure 3.**
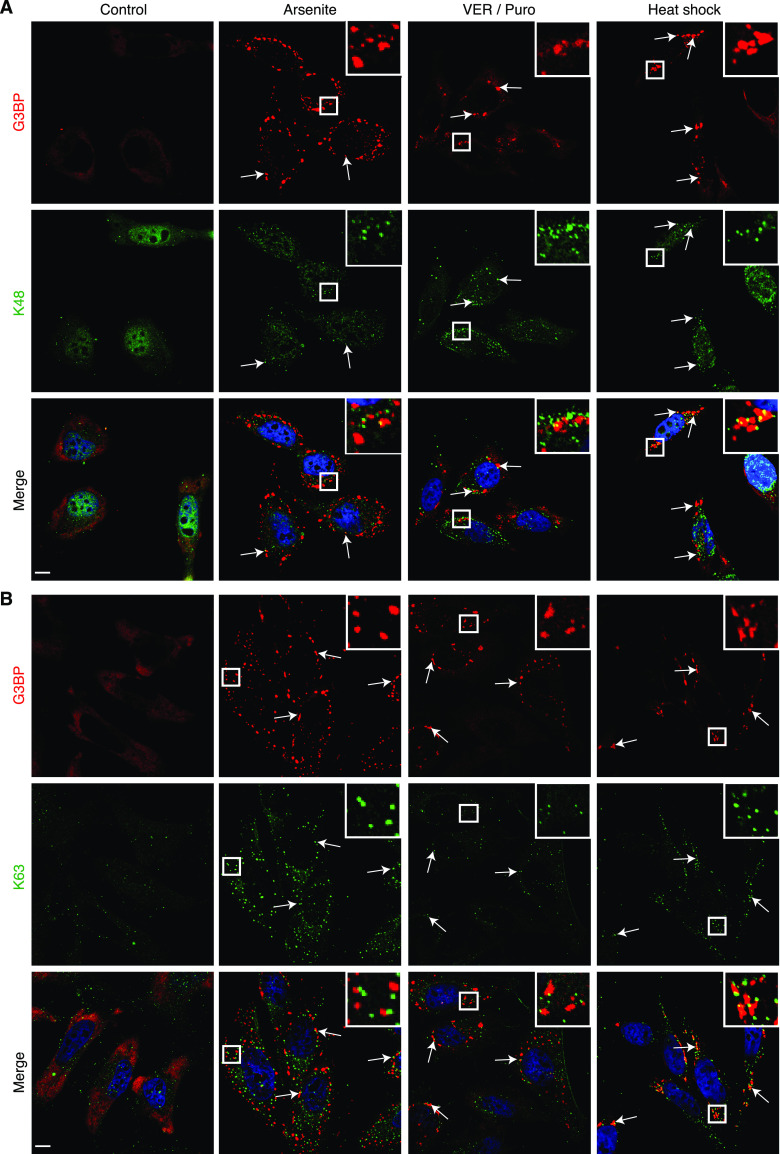
Stress granules contain K48- and K63-linked Ub chains. **(A, B)** HeLa cells were subjected to treatment with arsenite, VER/Puro and heat stress as indicated. **(A, B)** The colocalization between G3BP and K48-linked Ub chains (A) or K63-linked Ub chains (B) was visualized by confocal immunofluorescence microscopy. Representative Ub-positive stress granules are marked by arrows or magnified in the inset. Scale bar, 10 μm.

**Figure 4.**
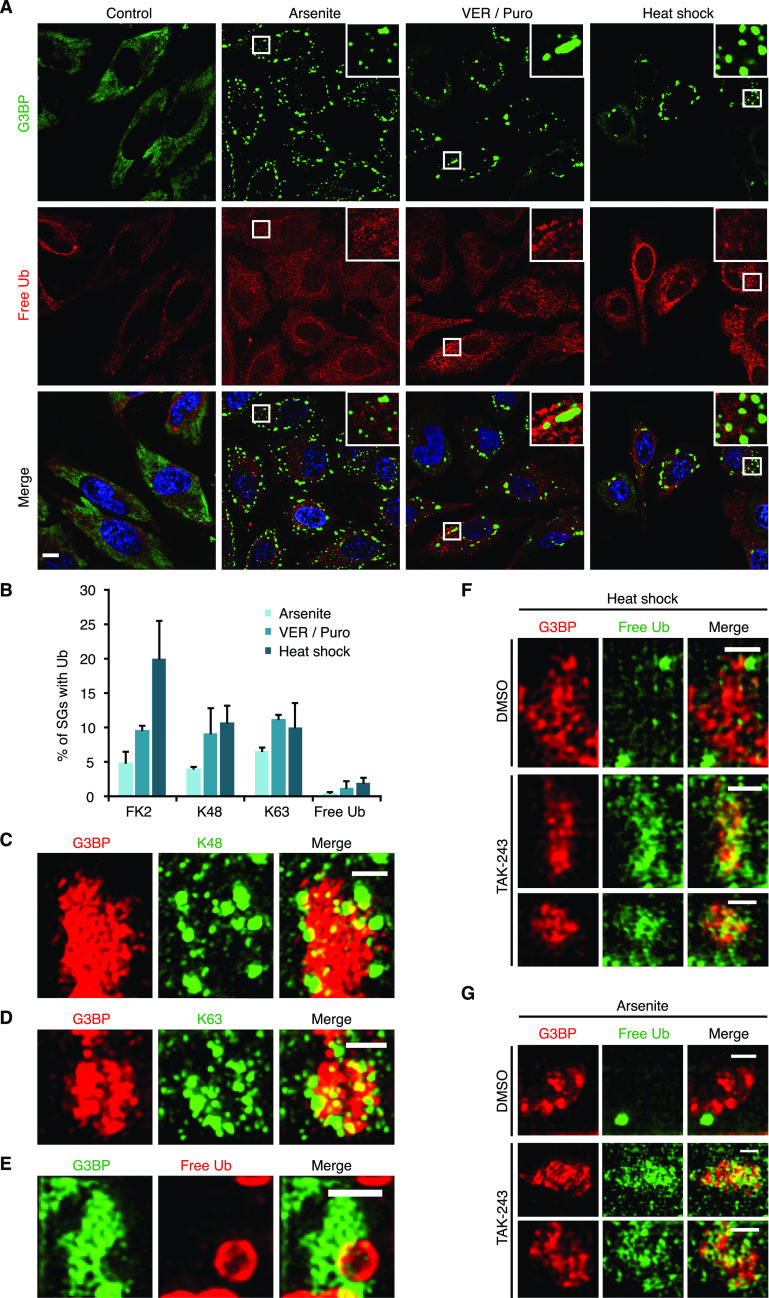
Stress granules contain Ub conjugates. **(A)** HeLa cells were subjected to treatment with arsenite, VER/Puro and heat stress as indicated. The colocalization between G3BP and free Ub was visualized by confocal immunofluorescence microscopy. Free Ub was detected using an antibody against the HA-tag of the free Ub sensor, HA-tUI. Representative Ub-positive SGs are magnified in the inset. Scale bar, 10 μm. **(A, B)** Quantification of Ub-positive SGs detected in (A) and in Fig 3A and B; shown is the mean ± SD; n = 2 with ≥1,000 SGs per condition. The data for FK2 from Fig 1C were included for comparison. **(C, D, E)** Structured illumination microscopy of heat stress-induced SGs positive for K48-linked Ub chains (C), K63-linked Ub chains (D), and free Ub (E). Scale bar, 1 μm. **(F)** HeLa cells were subjected to heat stress in the absence or presence of TAK-243 (1 μM) for 2 h. SGs and free Ub were stained using anti-G3BP and the free Ub sensor HA-tUI, respectively, followed by structured illumination microscopy. For TAK-243-treated cells, two representative images are shown. Scale bar, 1 μm. **(G)** HeLa cells were pre-treated with TAK-243 (1 μM, 1 h), followed by the addition of arsenite (0.5 mM) for 1 h in the continued presence of TAK-243. **(F)** SGs and free Ub were visualized as in (F). For TAK-243-treated cells, two representative images are shown. Scale bar, 1 μm.

To further characterize the Ub species present in SGs induced by arsenite, VER/Puro and heat shock, we performed immunofluorescence microscopy using monoclonal antibodies specific for K48- and K63-linked Ub chains, respectively, as well as a recently developed, avidity-based sensor for the detection of free Ub (HA-tUI) (Figs 3 and 4). HA-tUI binds to the free C-termini of mono-Ub and unanchored Ub chains with sub-nanomolar affinities and exhibits >10^6^-fold selectivity for free over conjugated Ub (Choi et al, 2019). The analysis with the linkage-specific antibodies revealed the presence of both K48- and K63-linked chains in all three types of SGs tested (Fig 3A and B), with relative frequencies similar to those determined with the FK2 antibody (Fig 4B). Whereas this is consistent with a previous report for heat- and VER/Puro-induced SGs (Xie et al, 2018), the association of K48- and K63-linked chains with arsenite-induced SGs had remained undetected in that study, perhaps because of their lower frequency. The recombinantly produced free Ub sensor HA-tUI was added to fixed and permeabilized cells and detected by anti-HA immunofluorescence microscopy (Choi et al, 2019). It showed a mostly diffuse cytoplasmic staining under control conditions, whereas additional cytoplasmic puncta were detectable under stress conditions (Fig 4A). However, these puncta were only rarely found in association with SGs, with a maximal frequency of 2% for heat-induced SGs, much lower than the frequencies observed for the K48- and K63-specific antibodies (Fig 4B).

**Figure S1.**
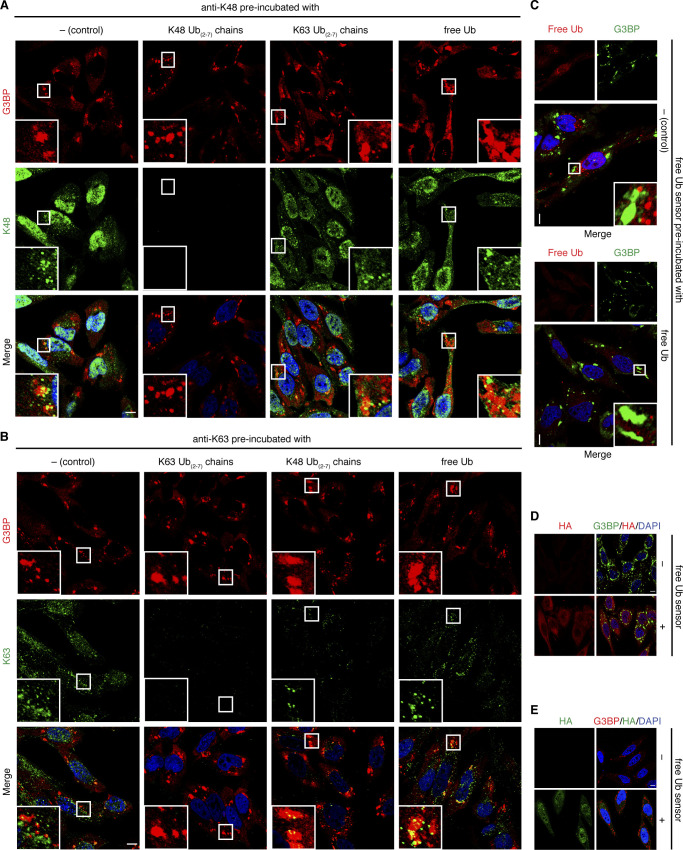
Specificity of anti-K48 and anti-K63 antibodies and of HA-tUI. **(A, B)** HeLa cells were subjected to heat stress (43°C, 2 h) and stained using anti-G3BP and anti-K48 (A) or anti-K63 (B) antibodies, followed by confocal immunofluorescence microscopy. The anti-K48/-K63 antibodies (1:100 dilution) were added either without preincubation (control), or after preincubation with K48-linked Ub chains, K63-linked Ub chains, or free mono-Ub (each at 100 μg/ml), as indicated. **(C)** HeLa cells were subjected to heat stress (43°C, 2 h), incubated with HA-tUI without preincubation (control) or after preincubation with a 1,000-fold molar excess of free mono-Ub, and stained using anti-G3BP and anti-HA antibodies, followed by confocal immunofluorescence microscopy. **(D, E)** HeLa cells were subjected to arsenite treatment (0.5 mM, 1 h), incubated with or without HA-tUI, and stained using anti-rabbit G3BP and anti-mouse HA antibodies (D) or anti-mouse G3BP and anti-rabbit HA antibodies (E), followed by confocal immunofluorescence microscopy. Scale bar, 10 μm. Source data are available for this figure.

Next, we confirmed that the specificities of the Ub detection reagents are maintained under the conditions of our immunofluorescence protocol. To that end, we preincubated the anti-K48 antibody with an excess of K48-linked Ub chains before its addition to heat-shocked, fixed cells. This preincubation completely eliminated the punctate, partially SG-associated immunofluorescence staining, whereas preincubation with K63-linked chains or free Ub failed to do so (Fig S1A). Similarly, blocking the K63-specific antibody with K63-linked Ub chains eliminated the punctate, partially SG-associated staining, whereas preabsorption with free Ub did not (Fig S1B). Preincubation of this antibody with K48-linked Ub chains resulted in a slight reduction in the anti-K63 immunofluorescence signal, indicating some cross-reactivity with K48-linked Ub chains. Finally, preincubation of HA-tUI with excess free mono-Ub (Fig S1C) or performing anti-HA immunofluorescence stainings in the absence of HA-tUI (Fig S1D and E) eliminated the punctate immunofluorescence staining, confirming the specificity of the free Ub sensor and the HA antibodies used.

We then used the three specific Ub detection reagents in SIM analyses of heat-induced SGs. The maximum intensity projections of SGs co-stained for G3BP and the K48- and K63-specific antibodies, respectively (Fig 4C and D), were very similar to those described above for the FK2 antibody (Fig 2C). These data indicate that the FK2 signals at SGs are largely derived from K48- and K63-linked Ub chains of at least two Ub moieties length, not from mono-ubiquitylated proteins. By contrast, the free Ub sensor HA-tUI stained distinct, circular structures of more than 0.5 μm diameter (Fig 4E), which sometimes overlapped with SGs, but never showed the characteristic, non-uniform intercalation into the cavities of the G3BP core observed with the anti-Ub antibodies. Although the identity of these structures remains unknown, they are clearly distinct from the SG-associated Ub chains stained by the FK2, anti-K48 and anti-K63 antibodies, strongly suggesting that these chains do not possess a free Ub C-terminus and, thus, do not represent unanchored chains.

**Figure S2.**
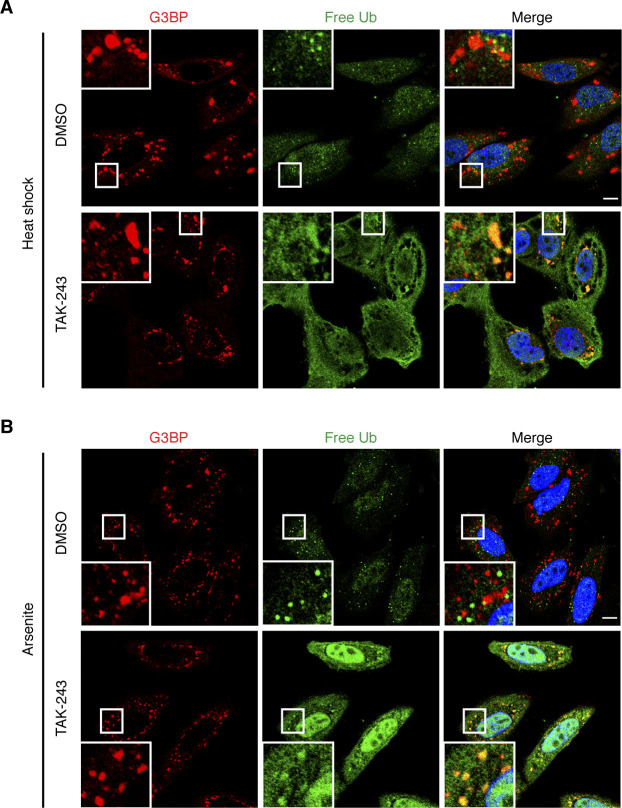
E1 inhibitor treatment increases the association of free Ub with stress granules. **(A)** HeLa cells were subjected to heat stress in the presence of TAK-243 (1 μM) for 2 h. SGs and free Ub were stained using anti-G3BP and anti-HA against free Ub sensor HA-tUI, followed by confocal immunofluorescence microscopy. **(B)** HeLa cells were pre-treated with TAK-243 (1 μM, 1 h), followed by the addition of arsenite (0.5 mM) for 1 h in the continued presence of TAK-243. **(A)** SGs and free Ub were visualized as in (A). Representative free Ub-positive SGs are magnified in the inset. Scale bar, 10 μm.

The absence of detectable amounts of free Ub and unanchored chains is in apparent conflict with two recent studies providing evidence for the presence of non-conjugated Ub at SGs (Xie et al, 2018; Markmiller et al, 2019). Because these studies relied on the use of ectopically expressed, tagged Ub variants, we wondered if the overexpression of Ub could have driven its reported association with SGs. To test this possibility, we increased the level of endogenous, unconjugated Ub by treatment with an inhibitor of the E1 Ub-activating enzyme, TAK-243 (also known as MLN7243), followed by SG induction. Intriguingly, upon TAK-243 treatment, HA-tUI no longer stained the large, circular structures described above. Instead, a more dispersed staining of the cavities and periphery of SGs was detected in SIM (Fig 4F and G) and confocal microscopy (Fig S2A and B), both for SGs induced by heat (Figs 4F and S2A) and arsenite (Figs 4G and S2B). We conclude that the previously reported association of non-conjugated Ub with SGs was likely promoted by unphysiologically high levels of free Ub due to overexpression.

In summary, our results show that both K48- and K63-linked Ub chains associate with SGs induced by different stress conditions, that these chains predominantly represent Ub conjugates rather than unanchored Ub chains, and that free mono-Ub at physiological concentrations is not a major SG-associated Ub species.

### p97 and the 26S proteasome co-localize with Ub at the periphery of SGs

**Figure S3.**
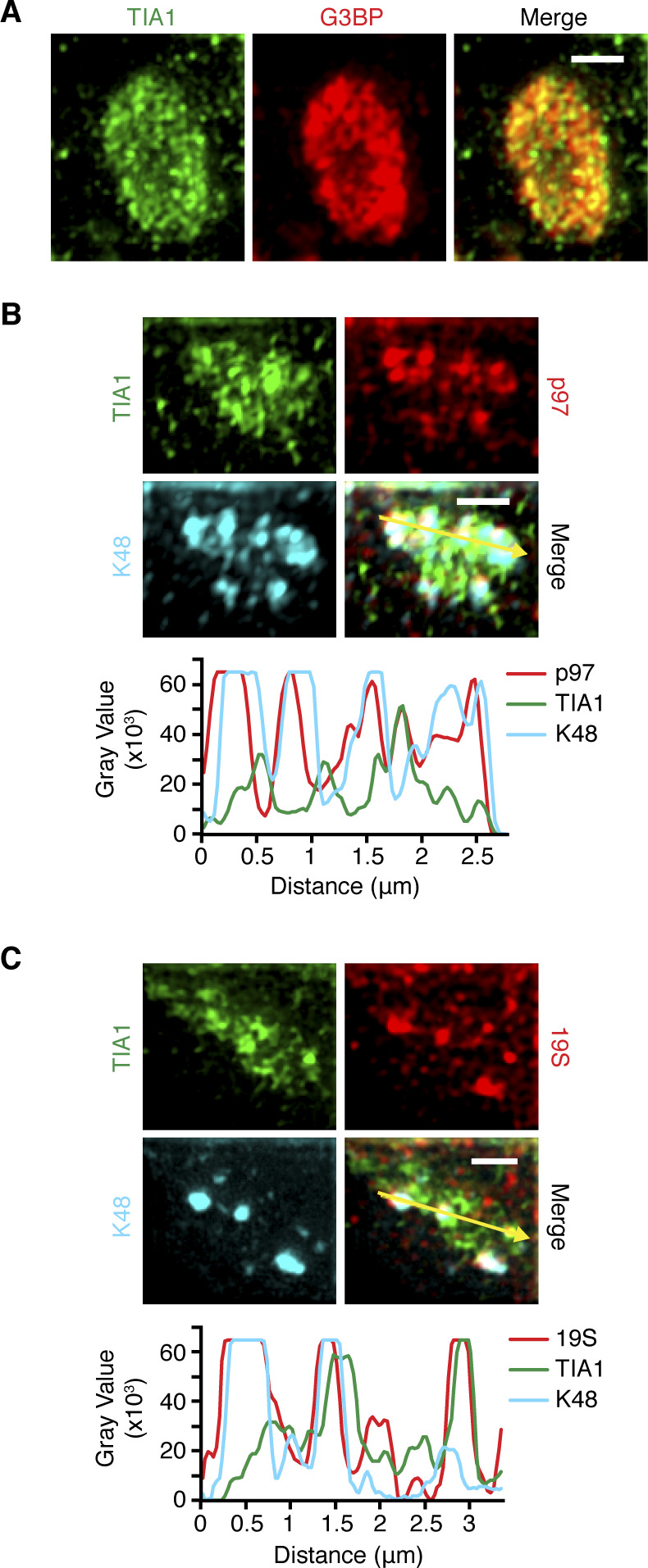
p97 and the 26S proteasome co-localize with Ub at the periphery of stress granules. **(A)** HeLa cells were subjected to arsenite stress (0.5 mM, 45 min) and stained with anti-G3BP and anti-TIA1, followed by structured illumination microscopy. Scale bar, 1 μm. **(B, C)** HeLa cells were subjected to heat stress (43°C, 2 h) and stained using anti-TIA1, anti-K48, and anti-p97 (B) or anti-Rpt6 (19S) antibodies (C), as indicated, followed by structured illumination microscopy. **(B, C)** Line profiles of TIA1 and K48 with p97 (B) or with 19S (C) along the yellow arrows were generated using ImageJ. Scale bar, 1 μm.

The presence of K48-linked Ub chains at SGs is consistent with the previously reported association of p97 and the 26S proteasome with SGs (Buchan et al, 2013; Turakhiya et al, 2018; Wang et al, 2019). To analyze the position of p97 and the 26S proteasome relative to Ub chains and the SG core at high resolution, we performed SIM on heat-induced SGs immunostained with antibodies against TIA1, K48-linked chains, as well as p97 and the 19S proteasomal subunit Rpt6 (also known as PSMC5, S8), respectively (Fig S3). The TIA1 antibody and a G3BP antibody co-stained the SG core, as expected (Fig S3A). Intriguingly, both p97 (Fig S3B) and the proteasome (Fig S3C) showed a significant colocalization with K48 chains at the periphery or intercalated into the cavities of the TIA1-positive SG core. These data support the conclusion that the K48-linked Ub chains at SGs represent covalent conjugates with target proteins, and they strongly suggest that the conjugates are subject to p97- and 26S proteasome-mediated degradation.

### SG association of ubiquitin chains is independent of the SUMO system

**Figure S4.**
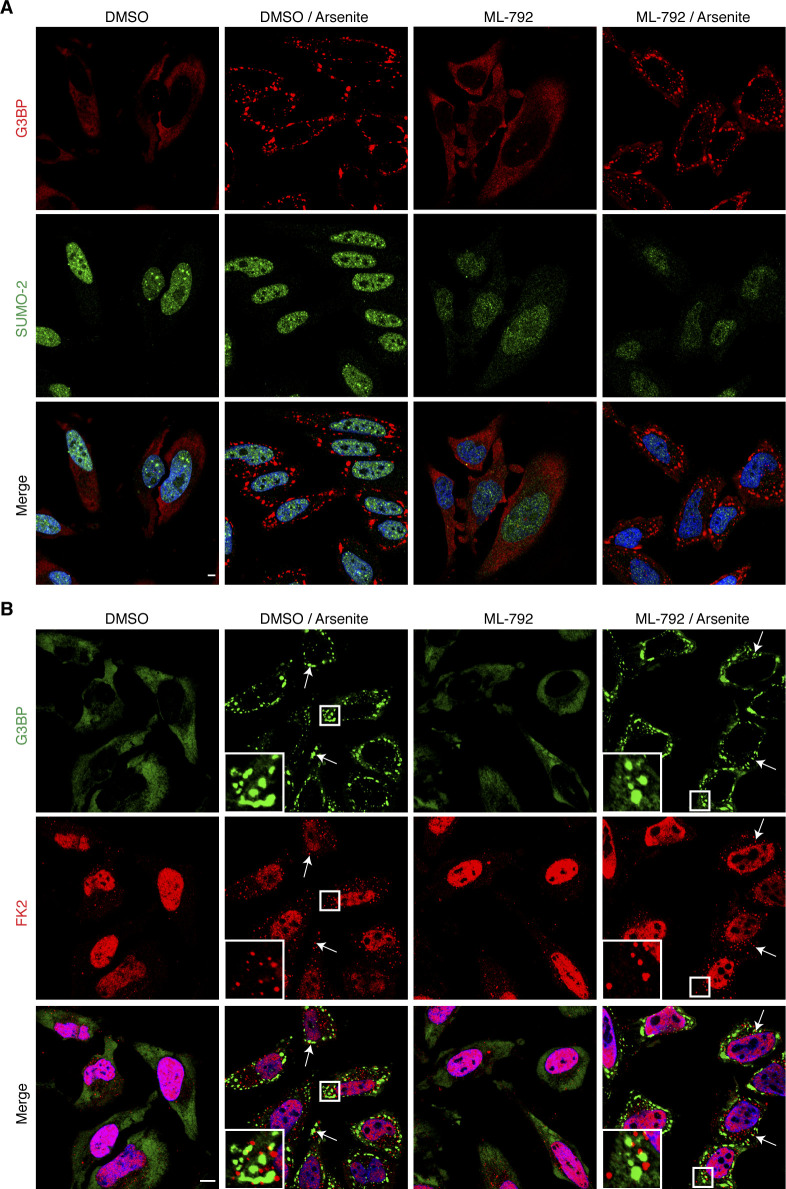
Ub conjugates at stress granules are independent of the SUMO system. **(A, B)** HeLa cells were pre-treated with ML-792 (1 μM, 1 h), followed by the addition of arsenite (0.5 mM) for 1 h in the continued presence of ML-792. **(A, B)** The cells were fixed and analyzed by confocal immunofluorescence microscopy using anti-G3BP and either anti-SUMO-2 (A) or anti-Ub (FK2; B) antibodies, respectively. In (B), representative FK2-positive stress granules are marked by arrows or magnified in the inset. Scale bar, 10 μm.

Very recently, the small ubiquitin-like modifier (SUMO) system and the SUMO-targeted Ub E3 ligase RNF4 were implicated in the process of SG clearance (Keiten-Schmitz et al, 2020; Marmor-Kollet et al, 2020). Therefore, we tested the possibility that the SG-associated Ub conjugates represent, at least in part, mixed SUMO-Ub chains. However, we were unable to detect any SUMO-2 signal at arsenite-induced SGs (Fig S4A), in agreement with a recent report (Keiten-Schmitz et al, 2020). Furthermore, inhibition of protein SUMOylation using the SUMO E1 inhibitor ML-792 reduced the predominantly nuclear SUMO-2 signal (Fig S4A), but did neither affect the formation of nor the Ub association with SGs (Fig S4A and B). These data clearly show that the association of Ub conjugates with SGs does not depend on the activity of the SUMO system.

### An active ubiquitin system is required for efficient SG clearance

**Figure 5.**
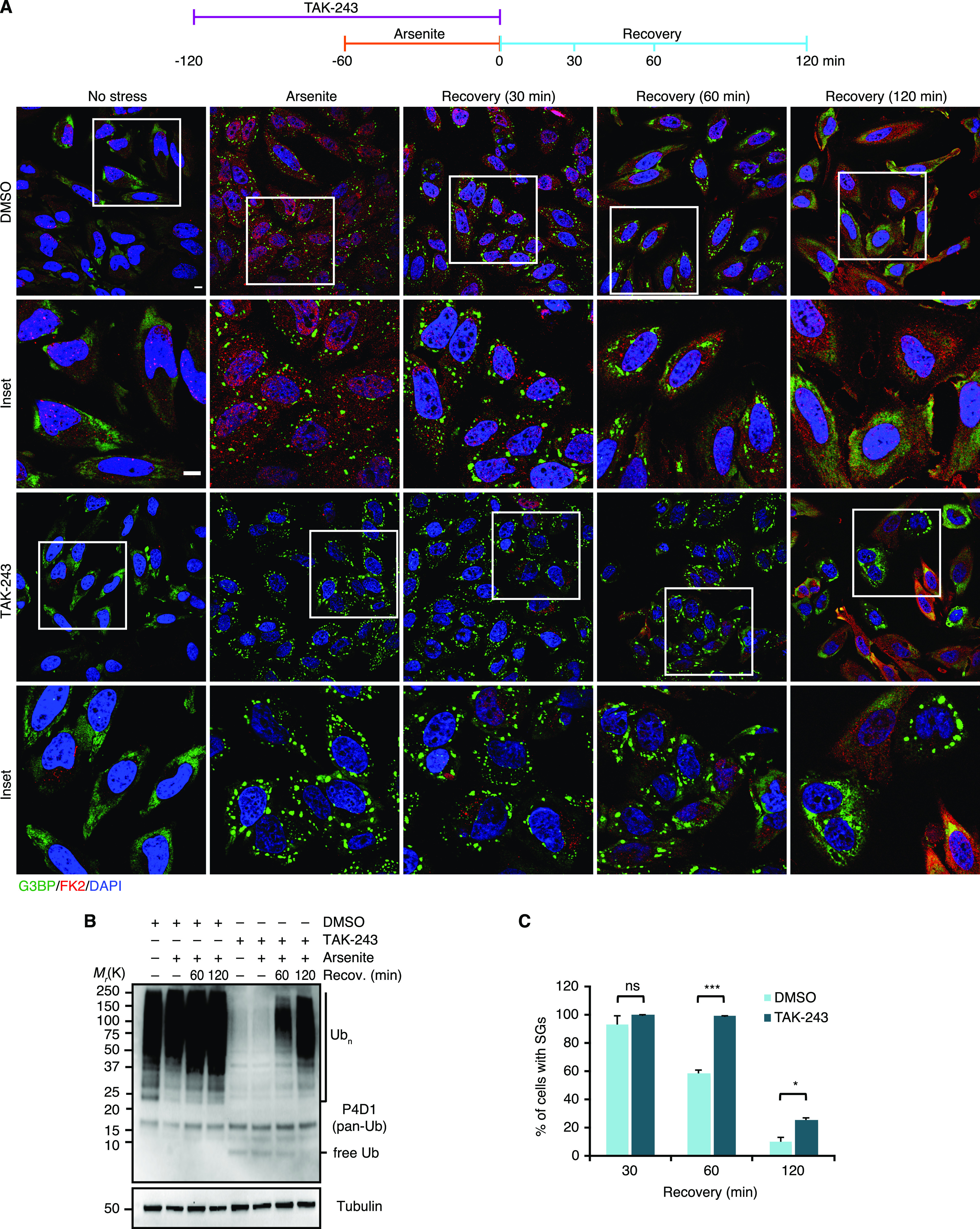
Active ubiquitylation is required for normal stress granule clearance. **(A)** HeLa cells were pre-treated with TAK-243 (1 μM, 1 h), followed by the addition of arsenite (250 μM) for 1 h in the continued presence of TAK-243. Subsequently, the cells were either fixed immediately or washed and allowed to recover under normal growth conditions over the indicated time course. SGs and Ub conjugates were visualized by confocal immunofluorescence microscopy using anti-G3BP and FK2 antibodies, respectively. Representative cells are magnified in the inset. Scale bar, 10 μm. **(A, B)** Immunoblot of whole-cell extracts from HeLa cells treated as in (A), using antibodies recognizing all forms of Ub (pan-Ub) or tubulin as loading control. **(A, C)** Quantification of cells with SGs in (A); shown is the mean ± SEM; n = 3 with ≥50 cells per replicate and condition; *P < 0.05; **P < 0.01; ***P < 0.001; ns, not significant; *t* test.

The results presented so far demonstrate the presence of Ub conjugates at various types of SGs. To analyze the functional relevance of the Ub system for granulostasis, we wished to determine the impact of E1 inhibition, initially by using an experimental setup that had been used in a previous study (Markmiller et al, 2019): Treatment with the Ub E1 inhibitor TAK-243 for 1 h, followed by SG induction with arsenite for 1 h in the continued presence of TAK-243, followed by washout of both TAK-243 and arsenite and recovery for up to 2 h in the absence of TAK-243 (see timeline, Fig 5A, top). Compared with the DMSO control, the TAK-243 treatment did not significantly affect the number and size of SGs formed upon arsenite stress (Fig 5A), even though the inhibitor efficiently prevented the formation of Ub chains and induced the accumulation of free mono-Ub (Fig 5B) (note that the almost complete loss of FK2 staining upon combined TAK-243 and arsenite treatment confirms the specificity of this antibody for Ub chains and conjugates [Fig 5A]). During recovery, however, the TAK-243-treated cells showed a significant delay in SG clearance, which was most pronounced after 60 min of recovery (Fig 5A and C). After 120 min of recovery, the difference between TAK-243-treated and control cells was still statistically significant, but smaller. Of note, by this time, the TAK-243-treated cells had started to produce Ub chains again (Fig 5B). Inspection of single cells in the microscopy images for this time point revealed that residual SGs were mainly present in cells with weak FK2 signal (Fig 5A), indicating that the smaller difference between TAK-243-treated and control cells after 120 min of recovery is most likely the consequence of an incipient loss of E1 inhibition. In summary, these data indicate that inhibition of the Ub system affects the clearance, but not the formation of arsenite-induced SGs.

**Figure 6.**
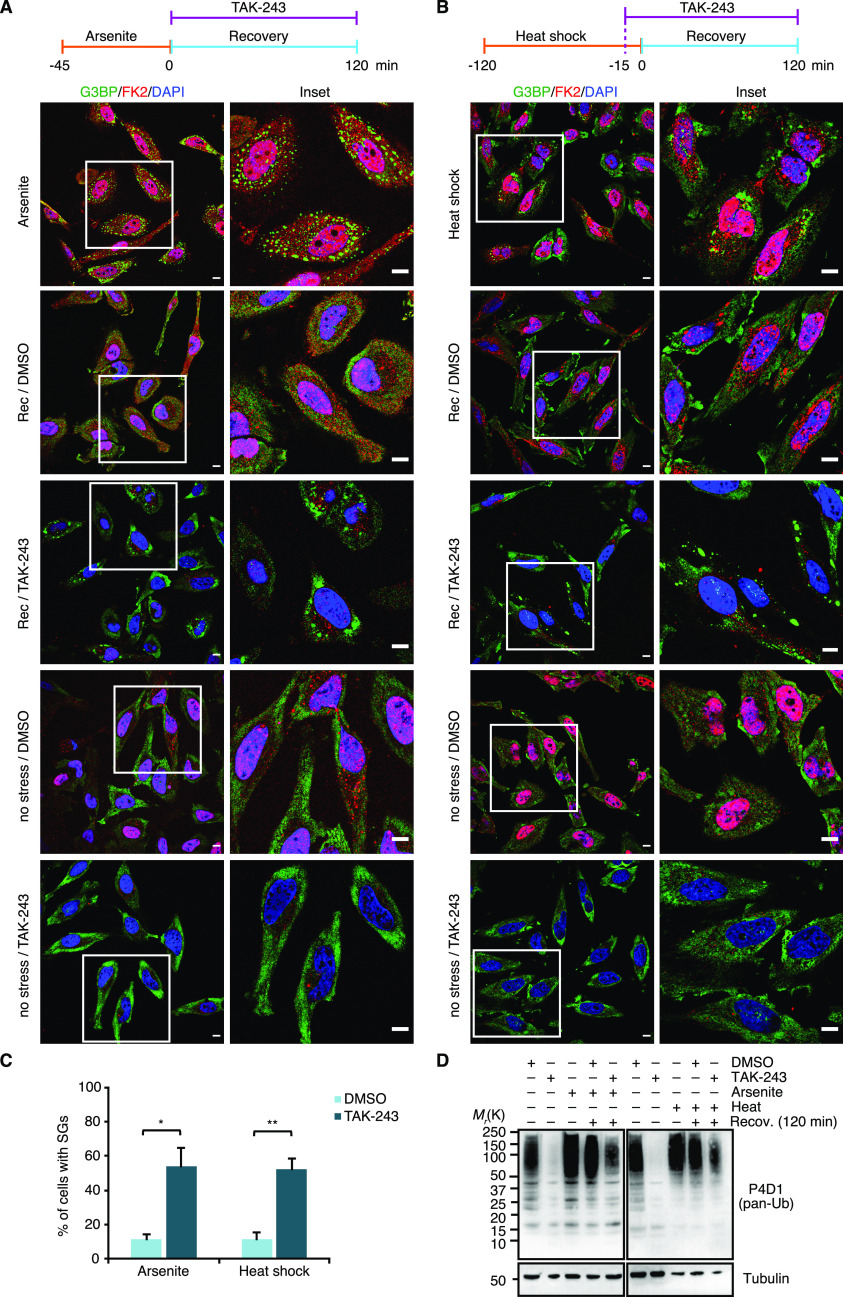
Active ubiquitylation is required during stress granule clearance. **(A)** HeLa cells were subjected to arsenite treatment (0.5 mM, 45 min), washed and allowed to recover in the presence of TAK-243 (1 μM) for 2 h, followed by confocal immunofluorescence microscopy using anti-G3BP and FK2 antibodies. Representative cells are magnified in the inset. Scale bar, 10 μm. **(B)** HeLa cells were subjected to heat stress (43°C, 2 h). 15 min before the end of the heat shock, TAK-243 (1 μM) was added, and the cells were subsequently allowed to recover at 37°C in the presence of TAK-243 for 2 h. Scale bar, 10 μm. **(A, B, C)** Quantification of cells with stress granules in (A) and (B); shown here is the mean ± SEM; n = 4 for (A) and n = 3 for (B) with ≥50 cells per replicate and condition; *P < 0.05; **P < 0.01; *t* test. **(A, B, D)** Immunoblot of whole-cell extracts from HeLa cells treated as in (A) and (B), using antibodies recognizing all forms of Ub (pan-Ub) or tubulin as loading control.

**Figure S5.**
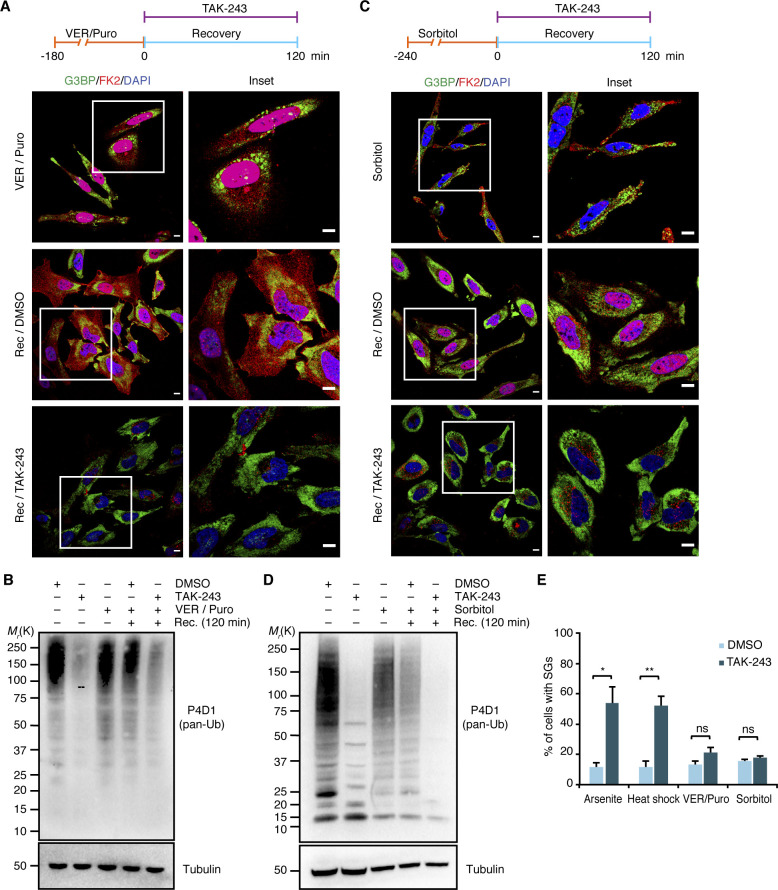
E1 inhibitor treatment has little effect on the clearance of VER/Puro- and sorbitol-induced stress granules. **(A, C)** HeLa cells were subjected to treatment with VER/Puro (3 h; A) or sorbitol (4 h; C), washed and allowed to recover in the presence of TAK-243 (1 μM) for 2 h, followed by confocal immunofluorescence microscopy using anti-G3BP and FK2 antibodies. Representative cells are magnified in the inset. Scale bar, 10 μm. **(A, B, C, D)** Immunoblot of whole-cell extracts from HeLa cells treated as in (A) or in (C), using antibodies recognizing all forms of Ub (pan-Ub) or tubulin as loading control. **(A, C, E)** Quantification of cells with stress granules in (A) and (C); shown here is the mean ± SEM; n = 3 with ≥ 50 cells per replicate and condition; *P < 0.05; **P < 0.01; ns, not significant; *t* test. The data for arsenite and heat stress from Fig 6C were included for comparison.

In the above experimental setup, the formal possibility remains that the pre-treatment with TAK-243 causes the accumulation of protein quality control (PQC) substrates before the addition of arsenite. This could result in the formation of SGs that contain elevated levels of misfolded proteins and are more difficult to clear for that very reason. To directly analyze the impact of the Ub system on the clearance of pre-formed SGs, we modified the experimental setup and added the E1 inhibitor *after* arsenite washout, right at the start of the recovery phase (Fig 6A). Intriguingly, after 2 h of recovery in the presence of TAK-243, more than 50% of the cells contained residual SGs, as compared with 10% in the absence of TAK-243 (Fig 6A and C). In a control experiment, treatment of cells with TAK-243 for 2 h without prior arsenite stress was indistinguishable from the DMSO control and did not result in any SG formation per se (Fig 6A, “no stress”). Remarkably, the strong effect of TAK-243 on the clearance of arsenite-induced SGs was observed even though the acute inhibitor treatment only partially blocked the formation of Ub conjugates under these conditions (Fig 6D). Very similar results were obtained for heat-induced SGs when TAK-243 was added 15 min before the heat-shocked cells were returned to 37°C for recovery (Fig 6B–D), whereas the clearance of SGs induced by VER/Puro or sorbitol was only slightly impaired without reaching statistical significance (Fig S5A, B, and E). We were unable to analyze the effect of the Ub system on the clearance of H_2_O_2_-induced SGs because the cells did not clear these SGs even in the absence of TAK-243 and upon recovery for up to 3 h, but underwent apoptosis instead (data not shown). Taken together, our results clearly demonstrate that an active Ub system is required for the normal clearance of SGs formed upon arsenite and heat stress.

### Pharmacological inhibition of ubiquitin conjugate turnover impairs SG clearance

**Figure 7.**
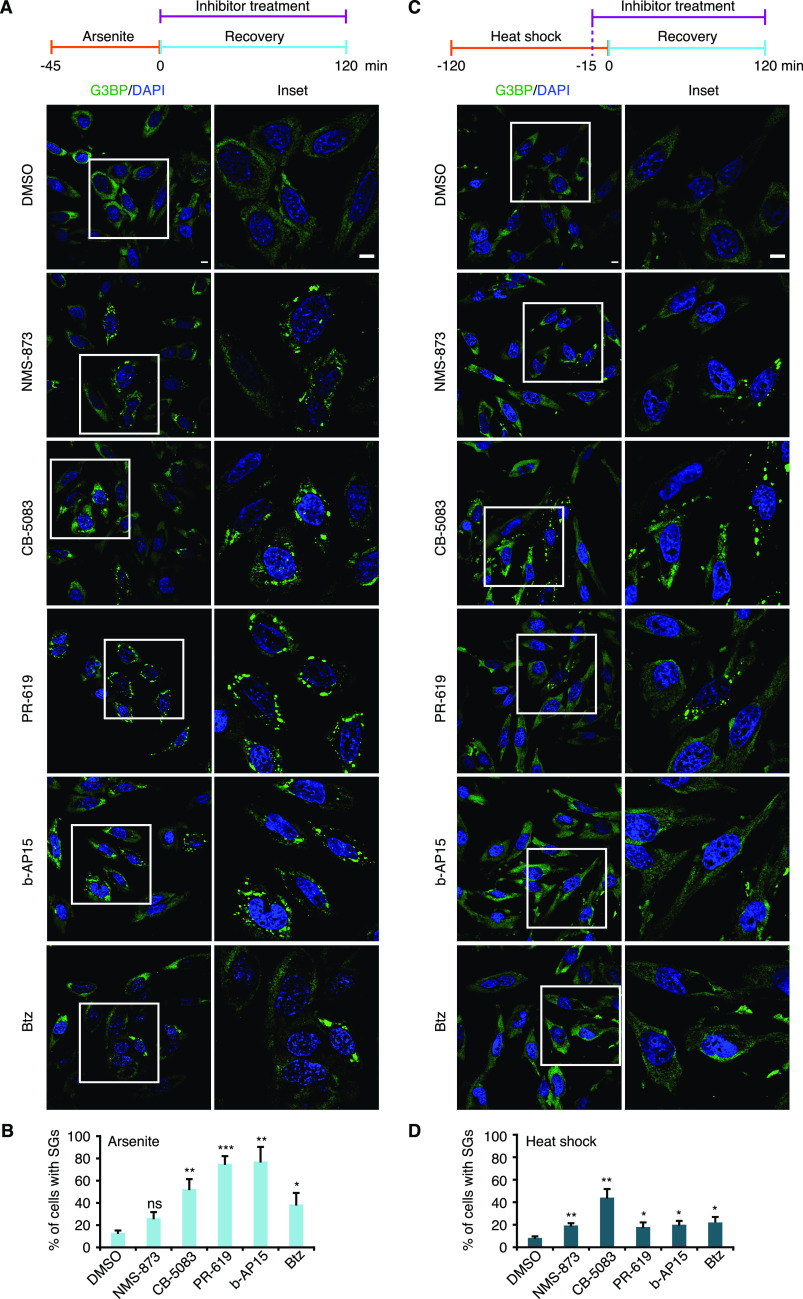
Turnover of Ub conjugates is required for efficient stress granule clearance. **(A)** HeLa cells were subjected to arsenite stress (0.5 mM, 45 min), washed, and subsequently allowed to recover in the presence of the indicated inhibitors for 2 h: NMS-873 (2.5 μM), CB-5083 (2.5 μM), PR-619 (5 μM), b-AP15 (0.5 μM), and Btz (1 μM). SGs were visualized by anti-G3BP confocal immunofluorescence microscopy. Scale bar, 10 μm. **(A, B)** Quantification of cells with SGs in (A); shown here is the mean ± SEM; n = 4 with ≥50 cells per replicate and condition; *P < 0.05; **P < 0.01; ***P < 0.001; ns, not significant; *t* test. **(C)** HeLa cells were subjected to heat stress (43°C, 2 h). **(A)** 15 min before the end of the heat shock, the same inhibitors as in (A) were added, and the cells were subsequently allowed to recover at 37°C in the presence of these inhibitors for 2 h. **(A)** Confocal immunofluorescence microscopy was performed as in (A). Scale bar, 10 μm. **(C, D)** Quantification of cells with SGs in (C); shown here is the mean ± SEM; n = 4 with ≥50 cells per replicate and condition. *P < 0.05; **P < 0.01; *t* test.

**Figure S6.**
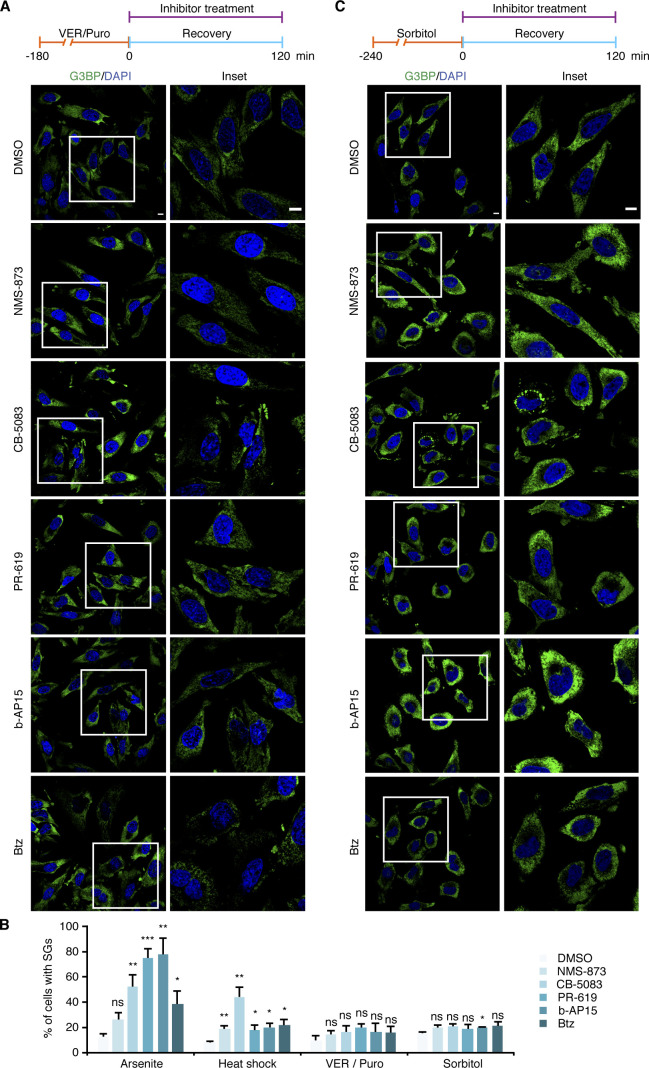
Ub system inhibitors have little effect on the clearance of VER/Puro- and sorbitol-induced stress granules. **(A, C)** HeLa cells were subjected to treatment with VER/Puro (3 h; A) or sorbitol (4 h; C), washed, and subsequently allowed to recover in the presence of the indicated inhibitors for 2 h: NMS-873 (2.5 μM), CB-5083 (2.5 μM), PR-619 (5 μM), b-AP15 (0.5 μM), and Btz (1 μM). SG-positive cells were determined by anti-G3BP confocal immunofluorescence microscopy. **(A, B)** Quantification of cells with SGs in (A) and (B); shown here is the mean ± SEM; n = 4 for (A) and n = 3 for (C) with ≥50 cells per replicate and condition; *P < 0.05; **P < 0.01; ***P < 0.001; ns, not significant; *t* test. The data for arsenite stress from Fig 7B and for heat stress from Fig 7D were included for comparison.

**Figure S7.**
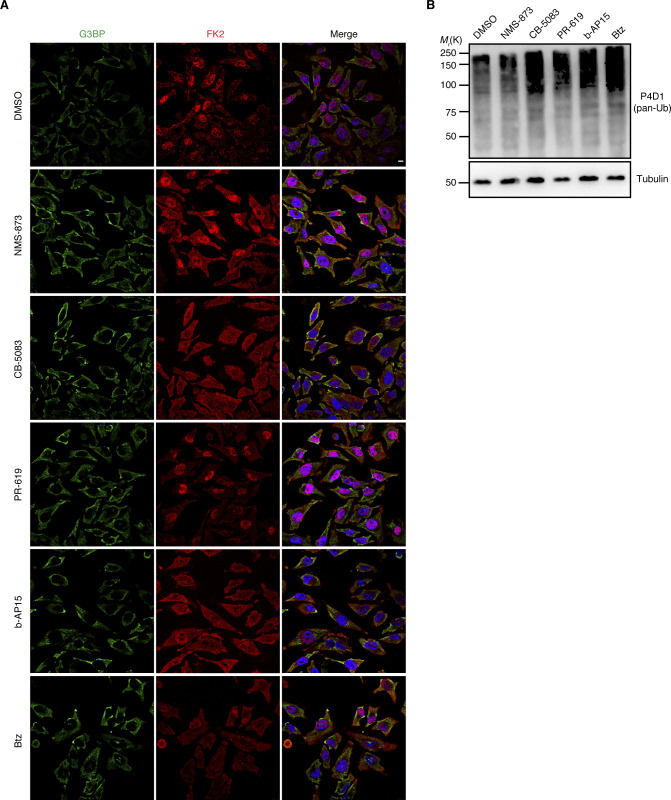
Ub system inhibitors moderately increase poly-Ub levels without inducing stress granule formation. **(A)** Unstressed HeLa cells were treated with indicated inhibitors for 2 h: NMS-873 (2.5 μM), CB-5083 (2.5 μM), PR-619 (5 μM), b-AP15 (0.5 μM), and Btz (1 μM). Stress granules and Ub were visualized by confocal immunofluorescence microscopy using anti-G3BP and FK2 antibodies, respectively. **(A, B)** Immunoblot of whole-cell extracts from HeLa cells treated as in (A), using antibodies recognizing all forms of Ub (pan-Ub) or tubulin as loading control.

The data so far showed that Ub conjugates are present at different types of SGs and that an active Ub system is required for the efficient clearance of some types of SGs. Next, we analyzed if the actual turnover of Ub conjugates is involved in SG clearance. To that end, we tested the impact of various chemical inhibitors of the ubiquitin proteasome system, including inhibitors of the 26S proteasome (Bortezomib, Btz), p97 (NMS-873; CB-5083), and DUBs (PR-619; b-AP15) (Fig 7). The inhibitors were added after SG induction by arsenite, heat, VER/Puro, and sorbitol, either immediately after inducer washout at the start of the 2-h recovery phase (arsenite, VER/Puro, and sorbitol), or 15 min before recovery (heat). Intriguingly, the clearance of arsenite-induced SGs was significantly impaired after acute inhibition of p97, DUBs, and the 26S proteasome, with the strongest effect seen with the general DUB inhibitor PR-619 and the proteasomal DUB inhibitor b-AP15 (Fig 7A and B). After 2 h of recovery, about 75% of the DUB inhibitor-treated cells still contained SGs, as compared with 10% for the DMSO vehicle control. The p97 inhibitor CB-5083 and the proteasome inhibitor Btz also caused a significant increase in the percentage of cells with residual SGs, whereas the increase observed with the p97 inhibitor NMS-873 did not reach statistical significance (Fig 7B). In the case of heat-induced SGs, the inhibitors also caused significant delays in SG clearance (Fig 7C and D), even though the effects were not as strong as with arsenite-induced SGs, with the two p97 inhibitors being more effective than the proteasome and DUB inhibitors. For VER/Puro- and sorbitol-induced SGs, the inhibitors caused slightly elevated percentages of cells with SGs after 2 h recovery which, however, did not reach statistical significance (Fig S6A–C). Importantly, treatment with the inhibitors for 2 h alone did not induce the formation of SGs (Fig S7A) but resulted in a moderate accumulation of Ub conjugates (Fig S7B). Our data, thus, link the delayed SG clearance observed in the presence of the inhibitors to the impaired turnover of ubiquitylated proteins. In summary, these results demonstrate that the turnover of ubiquitylated proteins is required for the normal clearance of SGs formed upon arsenite and heat stress.

## Discussion

RNP dynamics can be modulated by a variety of PTMs, including methylation, glycosylation, acetylation, phosphorylation, and SUMOylation (Hofweber & Dormann, 2019; Tauber et al, 2020; Hofmann et al, 2021). The present study substantiates a role of the Ub system in SG biology. It demonstrates that K48- and K63-linked Ub chains conjugated to substrate proteins are present at SGs induced by different stress conditions, and it strongly suggests that these Ub conjugates represent a major fraction of SG-associated Ub. Furthermore, it shows that active ubiquitylation, deubiquitylation and proteasomal turnover are necessary for the efficient clearance of two prototypic forms of SGs, that is, SGs induced by arsenite and heat shock.

Although there is considerable evidence in the literature for the association of Ub with SGs, the published data are heterogeneous with respect to the types of SG analyzed as well as the levels and identity of SG-associated Ub species reported (Kwon et al, 2007; Seguin et al, 2014; Mateju et al, 2017; Turakhiya et al, 2018; Xie et al, 2018; Markmiller et al, 2019; Zhang et al, 2019). Our confocal microscopy data show that up to 20% of heat-induced SGs can be stained with antibodies that are specific for Ub chains and mono-ubiquitylated substrates (FK2) and for K48- and K63-linked Ub chains, respectively (Fig 4B). Note that the frequencies of Ub-positive SGs were determined using single confocal planes to exclude the erroneous overlap of signals from distinct subcellular z levels. This approach could, in fact, result in an underestimation if additional Ub signals overlapped with the relatively large SGs outside of the confocal plane analyzed. Although this may account for the lower frequencies of Ub-positive SGs in comparison to some previous studies (Kwon et al, 2007; Seguin et al, 2014; Xie et al, 2018), our numbers for FK2-positive, heat-induced SGs are in excellent agreement with recent data obtained with the Ub chain–specific FK1 antibody (Mateju et al, 2017). The association of Ub with SGs induced by heat stress and VER/Puro has been consistently observed (Rodriguez-Ortiz et al, 2016; Mateju et al, 2017; Xie et al, 2018), whereas arsenite-induced SGs were either reported to be positive (Kwon et al, 2007; Seguin et al, 2014; Rodriguez-Ortiz et al, 2016; Turakhiya et al, 2018; Markmiller et al, 2019) or negative (Mateju et al, 2017; Xie et al, 2018) for Ub, and the Ub status of SGs induced by H_2_O_2_ and sorbitol has, to our knowledge, not been analyzed before. Our quantification shows that about 5% of arsenite-induced SGs are positive for Ub chains (Fig 1C). This relatively low frequency may explain why some previous studies failed to detect Ub at arsenite-induced SGs. Nevertheless, the association of Ub with arsenite-induced SGs is clearly supported by our super-resolution (SR) microscopy data (Fig 2) and is in line with the functional relevance of the Ub system for the clearance of arsenite-induced SGs (see below). By contrast, SGs induced by H_2_O_2_ and sorbitol were only rarely positive for Ub under our experimental conditions, suggesting a minor involvement of the Ub system.

Only few studies have so far addressed the identity of SG-associated Ub species and variably reported that they represent Ub conjugates (Mateju et al, 2017; Xie et al, 2018), unanchored Ub chains (Xie et al, 2018), or free mono-Ub (Markmiller et al, 2019). In the present study, the similar frequencies of Ub-positive SGs detected with the FK2, anti-K48-, and anti-K63 antibodies (Fig 4B) together with their similar structure in SR microscopy (Figs 2 and 4C and D) strongly suggest that the Ub signals originate from protein-conjugated or unanchored Ub chains, but not from mono-ubiquitylated proteins or free mono-Ub (which are not detected by the linkage-specific antibodies). Protein-conjugated and unanchored Ub chains, in turn, can be discriminated by virtue of the absence and presence, respectively, of a free Ub C-terminus. Our confocal and SIM analysis using the recombinant sensor protein HA-tUI, which has been shown before to stain endogenous free Ub in immunofluorescence microscopy (Choi et al, 2019), demonstrated a very low frequency (Fig 4B) and a distinct appearance (Fig 4G) of HA-tUI-positive structures at SGs, strongly suggesting that most of the SG-associated Ub chains exists in protein conjugates, not as unanchored chains.

Although our results did not provide evidence for a significant association of free Ub species with SGs, they do not exclude the possibility that a (small) subpopulation of unanchored Ub chains associates with SGs, as has recently been suggested for heat-induced SGs (Xie et al, 2018). By contrast, our data are in conflict with the recent proposal that arsenite-induced SGs co-localize primarily with free Ub (Markmiller et al, 2019). Of note, both studies used ectopic expression of tagged Ub variants to study the association of free Ub species with SGs. The interpretation of experiments using such Ub fusion proteins can, however, be complicated by both, non-physiological, elevated levels of the tagged Ub species and perturbations to the regulation of endogenous Ub. Indeed, we were able to show that non-conjugated Ub can be detected at arsenite- and heat-induced SGs if free Ub levels are elevated by E1 inhibition (Fig 4F and G). Our data suggest that the detection of free Ub species at SGs by Xie et al (2018) and Markmiller et al (2019) may be the consequence of artificially high levels of tagged, ectopically expressed Ub. Markmiller et al (2019) additionally performed immunofluorescence experiments using various Ub-specific antibodies (Markmiller et al, 2019), also including the FK2, anti-K48, and anti-K63 antibodies used and validated in our study. In contrast to our results, Markmiller et al (2019) reported that these antibodies stained a single, large perinuclear focus that did not overlap with SGs. We did not observe such an immunostaining under any condition tested (including prolonged arsenite stress for 120 min as in Markmiller et al (2019); data not shown), and the reason for this discrepancy remains unclear.

Our functional data obtained with small-molecule inhibitors targeting various enzymes of the Ub system confirm and extend previous reports implicating Ub-mediated protein turnover in granulostasis (Buchan et al, 2013; Turakhiya et al, 2018; Xie et al, 2018; Wang et al, 2019). Specifically, we found that the clearance of arsenite-induced SGs was strongly impaired by chemical inhibition of the Ub E1 enzyme, DUBs, the 26S proteasome, and p97 (Figs 5, 6A and C, and and 7A B), consistent with the recently proposed role of proteasomal proteolysis in this process (Turakhiya et al, 2018). Our data are thus in conflict with a recent study concluding that active protein ubiquitylation is dispensable for SG clearance, which was based on the lack of effects of E1 inhibition on the turnover of arsenite-induced SGs (Markmiller et al, 2019). However, upon closer inspection the data by Markmiller et al (2019) actually provide evidence for a delayed SG clearance upon E1 inhibition for HEK293T cells and, in one of several experiments, for HeLa S3 cells. It is possible that more pronounced clearance defects were obscured by the quantification method used by these authors, that is, calculating the total SG area of microscopy images relative to the total nuclear area, rather than quantifying the percentage of cells showing residual SGs. For the clearance of heat-induced SGs, E1 and p97 inhibitors had similar effects as with arsenite-induced SGs, whereas proteasomal and DUB inhibitors caused milder impairment (Figs 6B and C and 7B and C). By contrast, the inhibitors did not significantly impair the clearance of SGs induced by VER/Puro and sorbitol. Taken at face value, these results could indicate the existence of proteasome- and Ub system-independent clearance pathways for SGs induced by heat or VER/Puro and sorbitol, respectively. However, it has to be emphasized that the inhibitor treatment in these experiments was confined to the recovery phase to exclude potential confounding effects of inhibitor pre-treatment, such as accumulation of damaged proteins and/or depletion of Hsp70 chaperones and other proteostasis factors. Because the acute inhibitor treatment during recovery was only partially effective (Figs 6D, S5B and D, and S7 and B), our results may underestimate the true importance of the respective inhibitor targets and therefore do not necessarily exclude a contribution of proteasomal degradation and the Ub system to the efficient clearance of the respective SGs.

The differential effects of inhibitors of the Ub system on SG clearance reported here suggest that SGs have distinct requirements concerning the Ub system. This hypothesis is supported by the recent identification of proteins of the Ub system that are specifically involved in the clearance of certain types of SGs. The DUBs USP5 and USP13 are required for the normal assembly and clearance of SGs induced by heat, but not arsenite (Xie et al, 2018). Conversely, ZFAND1 is required for the efficient clearance of SGs induced by arsenite, but not other stressors (Turakhiya et al, 2018). The reason for the differential involvement of the Ub system is unclear. It may reflect differences in the proteotoxicity of the stressors, resulting in differing identities and/or levels of SG-associated PQC substrates, including defective ribosomal products (DRiPs) and misfolded proteins (Ganassi et al, 2016; Mateju et al, 2017; Turakhiya et al, 2018). Interestingly, a recent SIM analysis showed that misfolded mutant SOD1 forms distinct, non-homogenous structures at the periphery of SGs (Mateju et al, 2017) that are reminiscent of the Ub signals in our SIM images. Because mutant SOD1 is a short-lived substrate of the Ub proteasome system (Niwa et al, 2002), it is tempting to speculate that the Ub signals at SGs indeed represent ubiquitylated PQC substrates. However, our study shows that the frequency of ubiquitin conjugates at SGs is not strictly linked to the requirement for the Ub system during SG clearance (compare Figs 1C and S6B). Arsenite-induced SGs exhibit a low frequency of Ub association but strong dependency on the Ub system for clearance, whereas VER/Puro-induced SGs associate more frequently with Ub but hardly depend on the Ub system for clearance. Thus, the requirement for the Ub system in SG clearance does not simply reflect the amount of SG-associated Ub conjugates, but might depend on the actual folding/aggregation state and solubility of PQC substrates, as well as on the condensation state and dynamics of the SGs they associate with, under the respective stress condition.

Interestingly, the acute inhibition of the Ub E1 enzyme during recovery strongly impaired the clearance of both, arsenite- and heat-induced SGs (Fig 6). These results indicate that ongoing ubiquitylation is needed for the efficient clearance of these SGs, a requirement that cannot be explained by the turnover of PQC substrates or other pre-ubiquitylated proteins that had accumulated at SGs during stress treatment. In support of potential Ub system functions in SG clearance beyond PQC, it was recently shown that the optogenetically controlled multimerization of G3BP in the absence of any exogenous proteotoxic stress induces the formation of cytoplasmic granules (“OptoGranules”) that closely resemble classical SGs (Zhang et al, 2019). Importantly, these OptoGranules were found to be positive for Ub and to recruit Ub-binding proteins over time, raising the intriguing possibility that the Ub system is not merely eliminating SG-associated PQC substrates, but perhaps regulates the level or activity of proteins controlling SG dynamics. Such potential regulatory functions of the Ub system and the elimination of SG-associated PQC substrates described above need not be mutually exclusive, but could contribute to SG dynamics to varying degrees in dependence on the specific type of SG. For a deeper mechanistic understanding of the role of the Ub system in granulostasis, the future identification of endogenous target proteins as well as E3 ligases and DUBs controlling their ubiquitylation state will be instrumental.

## Materials and Methods

All materials used, including antibodies, proteins, and chemicals, are listed in Table S1.

Table S1. Materials used in this study.

### Mammalian cell culture

HeLa cells (CCL-2; ATCC) were cultured in DMEM supplemented with 10% fetal bovine serum and 1% penicillin/streptomycin in a humidified atmosphere with 5% CO_2_ at 37°C.

### SG induction and recovery

HeLa cells were seeded to 60% confluence on coverslips 24 h before stress treatment. To induce SG formation, the cells were subjected to one of the following stress conditions: sodium (meta)arsenite (0.5 mM, 45–60 min), puromycin (2.5 μg/ml) in combination with VER-155008 (40 μM, 3 h), sorbitol (0.4 M, 4 h), H_2_O_2_ (1 mM, 2 h), and heat shock (43°C, 2 h). After stress treatment, the cells were immediately harvested for immunofluorescence or allowed to recover for the indicated times either under normal growth conditions or in the presence of one of the following compounds: TAK-243 (1 μM), CB-5083 (2.5 μM), NMS-873 (2.5 μM), Btz (1 μM), PR-619 (5 μM), and b-AP15 (0.5 μM), followed by immunofluorescence as described below.

### Recombinant protein purification

Expression of HA-tUI from plasmid pET28a in *Escherichia coli* BL21 (DE3) pRIL was induced with 1 mM IPTG at 18°C overnight. The cells were harvested by centrifugation at 3,400*g* at 4°C, resuspended in ice-cold buffer A (20 mM sodium phosphate, pH 7.4, 500 mM NaCl, 10 mM imidazole, and 10 mM β-mercaptoethanol), and lysed using an EmulsiFlex-C5 High-Pressure Homogenizer (Avestin). The lysate was cleared by centrifugation at 45,000*g* for 30 min at 4°C and incubated with Ni-NTA agarose beads (QIAGEN) for 90 min at 4°C. The beads were washed twice with buffer A, and bound protein was eluted with 500 mM imidazole in buffer A. HA-tUI was further purified to homogeneity by gel filtration through a Superdex 75 HiLoad 26/60 column (GE Healthcare) in PBS (137 mM NaCl, 2.7 mM KCl, 10 mM Na_2_HPO_4_, and 1.8 mM KH_2_PO_4_, pH 7.4) supplemented with 1 mM DTT. Pure HA-tUI was concentrated, flash-frozen in liquid nitrogen, and stored at −80°C.

### Immunofluorescence

HeLa cells were washed twice with PBS, fixed using 3.7% formaldehyde in PBS for 15 min at RT, washed twice with cold PBS, and permeabilized with 0.2% Triton X-100 and 1% BSA in PBS for 30 min at RT. Cells were incubated with the indicated primary antibodies overnight at 4°C, washed twice with cold PBS, incubated with appropriate fluorophore-coupled secondary antibodies for 2 h at RT, and washed twice with cold PBS. Coverslips were mounted for microscopy with mounting medium containing 4′,6-diamidino-2-phenylindole (DAPI; Vectashield) and sealed with nail polish. To stain free ubiquitin/unanchored chains using the free ubiquitin sensor HA-tUI, cells were incubated after permeabilization with pure, recombinant HA-tUI (100 nM) for 30 min at RT, washed twice with cold PBS, followed by the incubation with a primary antibody directed against the HA epitope tag of HA-tUI. As a negative control, HA-tUI was preincubated with 100 μM free ubiquitin for 30 min at RT before addition to the cells. To confirm the specificity of the anti-K48 (Apu2) and anti-K63 (Apu3) anti-Ub antibodies, 1:100 dilutions of these antibodies were preincubated with 100 μg/ml of recombinant K48-linked Ub_(2-7)_ chains, K63-linked Ub_(2-7)_ chains, or free mono-Ub for 30 min at RT, followed by immunofluorescence.

### Microscopy and image processing

Confocal immunofluorescence microscopy was performed at the Imaging Core Facility (Biocenter, University of Würzburg) using a Leica TCS SP2 confocal microscope equipped with an acousto-optical beam splitter. Images were acquired using Leica confocal software. Imaging was performed using a 63×/1.4 oil objective and Diode UV (405 nm), Ar (488 nm), and HeNe (561 nm) lasers with three PMTs set to 407–470, 502–547, and 584–648 nm, respectively. Image processing was performed using Fiji (Schindelin et al, 2012). Representative images were processed using the brightness/contrast tool to adjust maximum display range without oversaturation, and using the background subtraction tool (rolling ball radius method).

SR microscopy of SGs was performed at the Imaging Core Facility (Biocenter, University of Würzburg) using a Zeiss Elyra S.1 SIM equipped with a PCO Edge 5.5 sCMOS camera. Image acquisition and processing were performed using the ZEN 2012 SP3 software (Zeiss). Imaging was conducted using a Plan-Apochromat 63×/1.4 oil objective, an HR Diode 488-100 nm laser with a BP495–550, LP750 emission filter, an HR DPSS 561-100 nm laser with a BP570–620, LP750 emission filter, and an HR Diode 642–150 nm laser with a LP655 emission filter. For Z-stack imaging, seven slices were captured at 400 nm Z-step size, followed by processing of SR-SIM images using the ZEN 2012 SP3 software. Maximum intensity projections of the Z-stack SR-SIM images were generated using Fiji.

### Quantification and statistical analysis

Quantification of SGs with ubiquitin was performed using the spot colocalization plugin ComDet v.0.4.1 by Fiji. Single plane images were used for all quantifications. Before running the plugin, every image was processed using a median filter to reduce noise, background subtraction (rolling ball radius method), and auto threshold (Renyi Entropy). SGs and Ub-positive foci were detected via the ComDet plugin using the following settings: approximated particle size was adjusted to 8 pixels for Ub and SG with intensity threshold (SD) of 3 pixels, larger particles were segmented during counting by the plugin, and the colocalization between SG and Ub was determined based on a maximum distance of 8 pixels between centroids of both structures. Quantifications of SGs with Ub were presented as the mean of two replicates ± SD, with at least 1,000 SGs per condition analyzed. The data were plotted using Excel (Microsoft Corporation).

Quantification of cells with SGs was performed by running the following macro in Fiji:

run("8-bit"); //for particle counting
run("Median...", "radius = 2");
run("Subtract Background...", "rolling = 10");
run("Enhance Contrast...", "saturated = 0 normalize");
run("Unsharp Mask...", "radius = 0 mask = 0.60");
//run("Brightness/Contrast...");
run("Auto Threshold", "method = Minimum white");
run("Set Measurements...", "area mean standard modal min centroid center perimeter bounding fit shape feret’s integrated median skewness kurtosis area_fraction stack display redirect = None decimal = 3");
run("Analyze Particles...", "size = 2–300 pixel show = Outlines exclude summarize add");
//to count particles

where the background was subtracted using the rolling ball radius method, and particles of size 2–300 pixels were analyzed using the analyze particle plugin. Regions of interest created by the above macro were used to count cells with SGs using the Fiji cell counter plugin. In addition, the total number of cells was counted using the Fiji cell counter plugin. Quantifications of cells with SGs are presented as the mean ± SEM, with at least 50 cells per replicate and condition. Statistical significance of differences between groups was evaluated using *t* test (two-tailed, unpaired). The normal distribution of the data was confirmed by performing the Shapiro–Wilk test using XLSTAT in Excel (Microsoft Corporation). Statistical analysis and generation of graphs were performed using Excel.

### Immunoblotting

For immunoblot analysis, cells were washed with PBS, resuspended in 1× Laemmli sample buffer (0.001% bromophenol blue, 10% glycerol, 2% SDS, 60 mM Tris–HCl, pH 6.8) supplemented with 100 mM DTT, and denatured at 95°C for 10 min. Proteins were resolved by electrophoresis on 4–20% Tris-Glycine gels (NuSep) in 1× MES running buffer, and transferred onto polyvinylidene fluoride (PVDF) membrane (Millipore) by tank blotting with 1× Tris-glycine buffer (192 mM glycine and 25 mM Tris-base, pH 8.3) supplemented with 20% methanol. The membrane was blocked with 5% milk in TBST (50 mM Tris–Cl, pH 7.5, 150 mM NaCl, and 0.1% Tween 20) and incubated with the indicated primary antibody in blocking solution overnight at 4°C. The membrane was washed with TBST (3 × 10 min), incubated with HRP-conjugated secondary antibody (Dianova) diluted 1:10,000 in blocking solution for 1 h at RT, washed again with TBST (3 × 10 min), and incubated with Clarity Western ECL Substrate (Bio-Rad). Chemiluminescence signals were detected using the Molecular Imager Gel Doc XR+ System (Bio-Rad). Immunoblot images were processed by Image Lab software (Bio-Rad).

## Supplementary Material

Reviewer comments
