# Supplementary material for: Comparative profiling of stress granule clearance reveals differential contributions of the ubiquitin system
Source: Life Sci Alliance. 2021 Mar 9;4(5):e202000927. doi: 10.26508/lsa.202000927 (PMC8008963; doi:10.26508/lsa.202000927)
Supplement: Supplementary file 2 [file LSA-2020-00927_TableS1.docx]

**Supplementary Table S1**

| **Reagent or resources** | **Source** | **Identifier** |
| --- | --- | --- |
| Antibodies |  |  |
| Alexa Fluor 488 Donkey Anti-Goat Ig (H+L) | Thermo Fisher Scientific | Cat# A11055 |
| Alexa Fluor 488 Goat Anti-Rabbit Ig (H+L) | Thermo Fisher Scientific | Cat# A11070 |
| Alexa Fluor 594 Goat Anti-Mouse Ig (H+L) | Thermo Fisher Scientific | Cat# A11070 |
| Alexa Fluor 594 Goat Anti-Rabbit Ig (H+L) | Thermo Fisher Scientific | Cat# A11072 |
| Alexa Fluor 647 Goat Anti-Rabbit Ig (H+L) | Thermo Fisher Scientific | Cat# A21246 |
| Anti-Mouse IgG (H+L) HRP | Dianova | Cat# 115-035-003 |
| Goat polyclonal anti-TIA1 | Sigma-Aldrich | Cat# SAB2501039 |
| Mouse monoclonal anti-G3BP1 | Abcam | Cat# ab56574 |
| Mouse monoclonal anti-HA tag | HISS Diagnostics GmbH | Cat# MMS-101R |
| Mouse monoclonal anti-mono- and polyubiquitinated conjugates (FK2) | Enzo Life Science | Cat# BML-PW8810-0500 |
| Mouse monoclonal anti-p97/VCP | Santa Cruz | Cat# sc-57492 |
| Mouse monoclonal anti-Rpt6 | Enzo Life Science | Cat# BML-PW9265-0025 |
| Mouse monoclonal anti-Ub (P4D1) | Enzo Life Science | Cat# BML-PW0930-0100 |
| Mouse monoclonal anti-α-Tubulin | Sigma-Aldrich | Cat# T5168 |
| Rabbit anti-SUMO-2 (Sentrin-2) | Thermo Fisher Scientific | Cat# 51-9100 |
| Rabbit monoclonal anti-HA tag | Cell Signaling Technology | Cat# 3724S |
| Rabbit monoclonal Lys48 specific anti-Ub (Apu2) | EMD Millipore | Cat# 05-1307 |
| Rabbit monoclonal Lys63 specific anti-Ub (Apu3) | EMD Millipore | Cat# 05-1308 |
| Rabbit polyclonal anti-G3BP1 | Thermo Fisher Scientific | Cat# PA5-29455 |
| Bacterial Strains |  |  |
| BL21 (DE3) pRIL | Agilent | Cat# 230245 |
| Chemicals and Recombinant Proteins |  |  |
| b-AP15 | EMD Millipore | Cat# 662140 |
| Bortezomib | Selleckchem | Cat# S1013 |
| Bovine Serum Albumin | Sigma-Aldrich | Cat# A9418 |
| CB-5083 | Selleckchem | Cat# S8101 |
| Clarity TM-Western ECL | Bio-Rad | Cat# 102031334 /102031336 |
| DMEM | Thermo Fisher Scientific | Cat# 41965_039 |
| DMSO | Thermo Fisher Scientific | Cat# D12345 |
| DTT | Carl Roth | Cat# 6908.2 |
| Fetal Bovine Serum | Thermo Fisher Scientific | Cat# 10270_106 |
| H_2_O_2_ | Merck | Cat# 107209 |
| Immobilon-P Transfer Membrane | Merck Millipore | Cat# IPVH00010 |
| K48-linked Poly-Ubiquitin (2-7) | Novus | Cat # UC-230-100 |
| K63-linked Poly-Ubiquitin (2-7) | Novus | Cat # UC-330-100 |
| ML-792 | MedChemExpress | Cat# HY-108702 |
| NaAsO_2_ | Sigma-Aldrich | Cat# S7400 |
| Ni-NTA Agarose | Qiagen | Catt# 30230 |
| NMS-873 | Selleckchem | Cat# S7285 |
| PBS | Thermo Fisher Scientific | Cat# 14190_094 |
| Penicillin-Streptomycin | Thermo Fisher Scientific | Cat# 15140_122 |
| PR-619 | EMD Millipore | Cat# 662141 |
| ProLong Glass Antifade Mountant | Thermo Fisher Scientific | Cat# P36980 |
| Puromycin | InvivoGen | Cat# ant-pr-1 |
| Sorbitol | Carl Roth | Cat# 6213.3 |
| TAK-243 | Selleckchem | Cat# S8341 |
| Tris-Glycine Precast Gel | NuSep | Cat# NG21-420 |
| Triton X-100 | Carl Roth | Cat# 3051.2 |
| Trypsin | Thermo Fisher Scientific | Cat# 25200_056 |
| Ubiquitin | Sigma-Aldrich | Cat# U6253 |
| Vectashield with DAPI | Vector Laboratories | Cat# H1200 |
| VER-155008 | Sigma-Aldrich/ Selleckchem | Cat# SML0271 /Cat# S7751 |
| Software and Algorithms |  |  |
| Fiji | <https://fiji.sc/> | PRID: SCR_00285 |
| Image Lab Software | Bio-Rad | RRID: SCR_014210 |
| ImageJ | <https://imagej.nih.gov/ij/> | PRID: SCR_003070 |
