# Supplementary material for: Comparative profiling of stress granule clearance reveals differential contributions of the ubiquitin system
Source: Life Sci Alliance. 2021 Mar 9;4(5):e202000927. doi: 10.26508/lsa.202000927 (PMC8008963; doi:10.26508/lsa.202000927)
Supplement: Reviewer comments [file LSA-2020-00927_review_history.pdf]

Comparative profiling of stress granule clearance reveals differential contributions of the ubiquitin system

Nazife Tolay and Alexander Buchberger
DOI: https://doi.org/10.26508/lsa.202000927

Corresponding author(s): Alexander Buchberger (University of Wuerzburg)

Review Timeline:

Submission Date:	2020-10-05
Editorial Decision:	2020-10-29
Revision Received:	2021-01-26
Editorial Decision:	2021-02-17
Revision Received:	2021-02-19
Accepted:	2021-02-23

Scientific Editor: Shachi Bhatt

Transaction Report:

(Note: With the exception of the correction of typographical or spelling errors that could be a source of ambiguity, letters and reports are not edited. The original formatting of letters and referee reports may not be reflected in this compilation.)

Please note that the manuscript was reviewed at Review Commons and these reports were taken into account in the decision-making process at Life Science Alliance.

No full Peer Review Process File is available with this article, as the authors have chosen not to make the review process public in this case.

October 29, 2020

Re: Life Science Alliance manuscript #LSA-2020-00927

Prof. Alexander Buchberger
University of Wuerzburg
Biochemistry
Biozentrum
Am Hubland
Wuerzburg 97074
Germany

Dear Dr. Buchberger,

Thank you for submitting your manuscript entitled "Comparative profiling of stress granule clearance reveals differential contributions of the ubiquitin system" to Life Science Alliance. We apologize for this unusual delay in getting back to you.

We would like to invite you to submit a revised manuscript to Life Science Alliance, in accordance to the revision plan that you laid out in the point-by-point response to the reviewers' comments.

As a brief overview, the manuscript was originally submitted and reviewed via Review Commons, after which the authors chose to submit the manuscript, reviewers' comments, and their points-by-point revision plan to address the reviewers' concerns to Life Science Alliance. The reviewers were enthusiastic about the findings, but pointed out some concerns that the authors plan to address in their revised manuscript.

To upload the revised version of your manuscript, please log in to your account: https://lsa.msubmit.net/cgi-bin/main.plex
You will be guided to complete the submission of your revised manuscript and to fill in all necessary information. Please get in touch in case you do not know or remember your login name.

We would be happy to discuss the individual revision points further with you should this be helpful.

While you are revising your manuscript, please also attend to the below editorial points to help expedite the publication of your manuscript. Please direct any editorial questions to the journal office.

The typical timeframe for revisions is three months. Please note that papers are generally considered through only one revision cycle, so strong support from the referees on the revised version is needed for acceptance.

When submitting the revision, please include a letter addressing the reviewers' comments point by point, and pointing out the changes in the revised manuscript.

Thank you for this interesting contribution to Life Science Alliance. We are looking forward to receiving your revised manuscript.

Sincerely,

Shachi Bhatt, Ph.D.
Executive Editor
Life Science Alliance
https://www.lsajournal.org/
Tweet @SciBhatt @LSAjournal

A. THESE ITEMS ARE REQUIRED FOR REVISIONS

-- A letter addressing the reviewers' comments point by point.

-- An editable version of the final text (.DOC or .DOCX) is needed for copyediting (no PDFs).

-- High-resolution figure, supplementary figure and video files uploaded as individual files: See our detailed guidelines for preparing your production-ready images, https://www.life-science-alliance.org/authors

-- Summary blurb (enter in submission system): A short text summarizing in a single sentence the study (max. 200 characters including spaces). This text is used in conjunction with the titles of papers, hence should be informative and complementary to the title and running title. It should describe the context and significance of the findings for a general readership; it should be written in the present tense and refer to the work in the third person. Author names should not be mentioned.

B. MANUSCRIPT ORGANIZATION AND FORMATTING:

Full guidelines are available on our Instructions for Authors page, https://www.life-science-alliance.org/authors

We encourage our authors to provide original source data, particularly uncropped/-processed electrophoretic blots and spreadsheets for the main figures of the manuscript. If you would like to add source data, we would welcome one PDF/Excel-file per figure for this information. These files will be linked online as supplementary "Source Data" files.

IMPORTANT: It is Life Science Alliance policy that if requested, original data images must be made available. Failure to provide original images upon request will result in unavoidable delays in publication. Please ensure that you have access to all original microscopy and blot data images before submitting your revision.

February 17, 2021

RE: Life Science Alliance Manuscript #LSA-2020-00927R

Prof. Alexander Buchberger
University of Wuerzburg
Biochemistry
Biozentrum
Am Hubland
Wuerzburg 97074
Germany

Dear Dr. Buchberger,

Thank you for submitting your revised manuscript entitled "Comparative profiling of stress granule clearance reveals differential contributions of the ubiquitin system". We would be happy to publish your paper in Life Science Alliance pending final revisions necessary to meet the reviewers' minor requests and our formatting guidelines.

Along with the points listed below, please also attend to the following,

-please add a callout for Figures 2A, B, E; S5E, and S6C to your main manuscript text
-please use the [10 author names, et al.] format in your references (i.e. limit the author names to the first 10)
-please revise the inset position in Figure 1A arsenite row FK2 column; and row H2O2 FK2 column; Figure S1 first row Freeub column so that they match the zoomed in parts
-please add scale bar to figure 3, 4A, F, G
-please re-position the scale bars in Fig 2, Fig 4C, D, E, Fig S3A, B, C, such that they are more visible
-please provide unedited source data for all the panels in Fig S1A

If you are planning a press release on your work, please inform us immediately to allow informing our production team and scheduling a release date.

To upload the final version of your manuscript, please log in to your account: https://lsa.msubmit.net/cgi-bin/main.plex
You will be guided to complete the submission of your revised manuscript and to fill in all necessary information. Please get in touch in case you do not know or remember your login name.

To avoid unnecessary delays in the acceptance and publication of your paper, please read the following information carefully.

A. FINAL FILES:

These items are required for acceptance.

-- An editable version of the final text (.DOC or .DOCX) is needed for copyediting (no PDFs).

-- High-resolution figure, supplementary figure and video files uploaded as individual files: See our detailed guidelines for preparing your production-ready images, https://www.life-science-alliance.org/authors

-- Summary blurb (enter in submission system): A short text summarizing in a single sentence the study (max. 200 characters including spaces). This text is used in conjunction with the titles of papers, hence should be informative and complementary to the title. It should describe the context and significance of the findings for a general readership; it should be written in the present tense and refer to the work in the third person. Author names should not be mentioned.

B. MANUSCRIPT ORGANIZATION AND FORMATTING:

Full guidelines are available on our Instructions for Authors page, https://www.life-science-alliance.org/authors

We encourage our authors to provide original source data, particularly uncropped/-processed electrophoretic blots and spreadsheets for the main figures of the manuscript. If you would like to add source data, we would welcome one PDF/Excel-file per figure for this information. These files will be linked online as supplementary "Source Data" files.

Submission of a paper that does not conform to Life Science Alliance guidelines will delay the acceptance of your manuscript.

It is Life Science Alliance policy that if requested, original data images must be made available to the editors. Failure to provide original images upon request will result in unavoidable delays in publication. Please ensure that you have access to all original data images prior to final submission.

The license to publish form must be signed before your manuscript can be sent to production. A link to the electronic license to publish form will be sent to the corresponding author only. Please take a moment to check your funder requirements.

Reviews, decision letters, and point-by-point responses associated with peer-review at Life Science Alliance will be published online, alongside the manuscript. If you do want to opt out of having the reviewer reports and your point-by-point responses displayed, please let us know immediately.

Thank you for your attention to these final processing requirements. Please revise and format the manuscript and upload materials within 7 days.

Thank you for this interesting contribution, we look forward to publishing your paper in Life Science Alliance.

Sincerely,

Shachi Bhatt, Ph.D.
Executive Editor
Life Science Alliance
https://www.lsajournal.org/
Tweet @SciBhatt @LSAjournal
Interested in an editorial career? EMBO Solutions is hiring a Scientific Editor to join the international Life Science Alliance team. Find out more here - https://www.embo.org/documents/jobs/Vacancy_Notice_Scientific_editor_LSA.pdf

February 23, 2021

RE: Life Science Alliance Manuscript #LSA-2020-00927RR

Prof. Alexander Buchberger
University of Wuerzburg
Biochemistry
Biozentrum
Am Hubland
Wuerzburg 97074
Germany

Dear Dr. Buchberger,

Thank you for submitting your Research Article entitled "Comparative profiling of stress granuleclearancerevealsdifferentialcontributionsoftheubiquitinsystem". It is a pleasure to let you know that your manuscript is now accepted for publication in Life Science Alliance. Congratulations on this interesting work.

The final published version of your manuscript will be deposited by us to PubMed Central upon online publication.

Your manuscript will now progress through copyediting and proofing. It is journal policy that authors provide original data upon request.

Reviews, decision letters, and point-by-point responses associated with peer-review at Life Science Alliance will be published online, alongside the manuscript. If you do want to opt out of having the reviewer reports and your point-by-point responses displayed, please let us know immediately.

IMPORTANT: If you will be unreachable at any time, please provide us with the email address of an alternate author. Failure to respond to routine queries may lead to unavoidable delays in publication.

Scheduling details will be available from our production department. You will receive proofs shortly before the publication date. Only essential corrections can be made at the proof stage so if there are any minor final changes you wish to make to the manuscript, please let the journal office know now.

DISTRIBUTION OF MATERIALS:
Authors are required to distribute freely any materials used in experiments published in Life Science Alliance. Authors are encouraged to deposit materials used in their studies to the appropriate repositories for distribution to researchers.

You can contact the journal office with any questions, contact@life-science-alliance.org

Again, congratulations on a very nice paper. I hope you found the review process to be constructive and are pleased with how the manuscript was handled editorially. We look forward to future exciting submissions from your lab.

Sincerely,

Shachi Bhatt, Ph.D.
Executive Editor
Life Science Alliance
https://www.lsajournal.org/
Tweet @SciBhatt @LSAjournal
Interested in an editorial career? EMBO Solutions is hiring a Scientific Editor to join the international Life Science Alliance team. Find out more here - https://www.embo.org/documents/jobs/Vacancy_Notice_Scientific_editor_LSA.pdf
